# TIPIN Couples Epigenetic Rewiring to EGFR Stabilization and Lenvatinib Resistance in Liver Cancer

**DOI:** 10.1002/advs.78638

**Published:** 2026-11-09

**Authors:** Xing Jia, Shiyu Zhang, Yangbo Tang, Shinuo Chen, Xingxin Zhu, Junran Chen, Suchen Bian, Lei Zheng, Chi Zhang, Haojing Ma, Wengfeng Song, Cheng Jin, Mengqiao Zhou, Haiyang Xie, Qinglin Li, Penghong Song

**Affiliations:** ^1^ Division of Hepatobiliary and Pancreatic Surgery Department of Surgery The First Affiliated Hospital Zhejiang University School of Medicine Hangzhou China; ^2^ Thyroid Surgery Department Zhejiang Cancer Hospital Hangzhou Zhejiang China; ^3^ NHC Key Laboratory of Combined Multi‐Organ Transplantation Hangzhou China; ^4^ Key Laboratory of the Diagnosis and Treatment of Organ Transplantation Research Unit of Collaborative Diagnosis and Treatment for Hepatobiliary and Pancreatic Cancer Chinese Academy of Medical Sciences (2019RU019) Hangzhou China; ^5^ Key Laboratory of Organ Transplantation Research Center for Diagnosis and Treatment of Hepatobiliary Diseases Hangzhou Zhejiang China; ^6^ Department of Thoracic Surgery Second Affiliated Hospital Zhejiang University School of Medicine Hangzhou China; ^7^ Department of Thoracic Surgery The First Affiliated Hospital of Zhejiang University School of Medicine Hangzhou China; ^8^ Tongde Hospital of Zhejiang Province Affiliated to Zhejiang Chinese Medical University Hangzhou Zhejiang China; ^9^ State Key Laboratory for Diagnosis and Treatment of Infectious Diseases First Affiliated Hospital Zhejiang University School of Medicine Hangzhou China

**Keywords:** EGFR, lenvatinib resistance, liver cancer, TIPIN, ubiquitination

## Abstract

**Aims**: Liver cancer is an aggressive tumor marked by substantial morbidity and mortality. Timeless‐interacting protein (TIPIN) is dysregulated in multiple cancers, but its epigenetic regulation, biological function, and downstream pathways in liver cancer remain poorly defined. This study sought to explore TIPIN's function in liver cancer tumorigenesis, metastasis, and lenvatinib resistance.

**Methods**: We analyzed TCGA data and 94 paired clinical samples. DNA methylation sequencing, whole‐body TIPIN knockout mouse hydrodynamic injection models, RNA‐seq, immunoprecipitation‐mass spectrometry, co‐immunoprecipitation, protein stability and ubiquitination assays, and patient‑derived xenograft and patient‑derived organoid models were used.

**Results**: TIPIN was upregulated by DNA hypomethylation in liver cancer and associated with poor prognosis. TIPIN promoted liver cancer initiation and progression in vivo. Mechanistically, TIPIN binds Epidermal Growth Factor Receptor (EGFR) and competes with E3 ligase c‑Cbl for the Tyr1045‐containing interface, thereby repressing c‑Cbl‐mediated EGFR ubiquitination and degradation and activating downstream ERK1/2 signaling to facilitate tumor proliferation and metastasis. TIPIN depletion accelerates EGFR turnover and sensitizes liver cancer cells to lenvatinib.

**Conclusions**: TIPIN stabilizes EGFR via competitive binding to the Tyr1045 interface, restricting its ubiquitin‐dependent degradation to drive liver cancer progression and lenvatinib resistance. Targeting the TIPIN–EGFR axis could be an effective therapy for liver cancer.

## Graphical abstract

DNA hypomethylation elevates TIPIN expression in liver cancer. TIPIN competes with E3 ligase c‐Cbl for the EGFR intracellular domain, inhibiting EGFR ubiquitination and lysosomal degradation to sustain oncogenic EGFR‐ ERK signaling. High TIPIN confers lenvatinib resistance, while TIPIN depletion restores drug sensitivity in liver cancer.

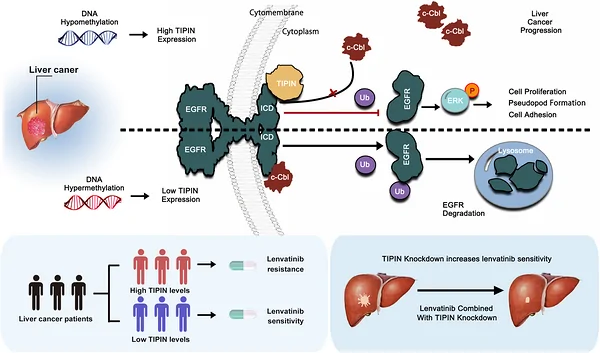

## 1 Introduction

Liver cancer represents one of the leading causes of cancer‐related death worldwide. This high mortality is driven primarily by underlying cirrhosis, late clinical presentation, and limited efficacy of available systemic therapies [1]. Over the past decade, systemic treatment options have expanded with the introduction of tyrosine kinase inhibitors (TKIs) [2, 3]. Immune checkpoint inhibitor‑based combinations targeting programmed death‑1 programmed death‑ligand 1 and Vascular Endothelial Growth Factor (VEGF) pathways have also entered routine use [4], leading to modest improvements in survival. For patients with early‐stage disease, surgical resection and local ablative therapies are the main treatment options [5, 6]. Nevertheless, disease recurrence after curative therapy and the development of resistance to systemic treatments remain major clinical challenges [7]. Therefore, there is an urgent need to unravel the molecular basis of drug resistance in treatment to improve the prognosis of liver cancer patients.

Dysregulated epidermal growth factor receptor (EGFR) signaling cascade is a recurrent molecular feature of liver cancer and plays a causal role in tumor malignant progression [8, 9]. EGFR overexpression and downstream activation of the mitogen‐activated protein kinase (MAPK) pathway are associated with invasive tumor phenotypes and unfavorable prognosis [10]. Clinical trials of EGFR inhibitors in unselected liver cancer populations have yielded disappointing results [11, 12]. Moreover, resistance to first‐line TKIs such as lenvatinib may involve adaptive activation of the EGFR axis and altered receptor turnover [13, 14]. Therefore, revealing the regulatory mechanisms that control the stability and degradation of EGFR proteins may lead to the discovery of new strategies to avoid therapeutic resistance.

Epigenetic reprogramming is recognized as a decisive sign of liver cancer, impacting every hallmark of tumorigenesis including malignant transformation, immune evasion, and therapeutic response [15, 16, 17]. The liver cancer epigenome is profoundly remodeled by genome‐wide alterations in DNA methylation—characterized by overall hypomethylation of tumor suppressor genes and hypermethylation of focal promoters [18, 19]. DNA methyltransferases are frequently upregulated in liver cancer, while TET‐family demethylases are often downregulated [20, 21]. Identifying epigenetically regulated genes that control receptor stability and signaling holds great promise for therapeutic opportunities [16, 22, 23].

The turnover of EGFR protein is controlled by both ubiquitin‐dependent and noncanonical degradation pathways [8]. EGFR undergoes dimerization, autophosphorylation, and recruitment of the c‐Cbl via phosphotyrosine Y1045, triggering polyubiquitination, endocytosis, multivesicular body formation, and lysosomal degradation [24, 25, 26]. In contrast, ligand‐independent [27] or stress‐induced EGFR degradation [28] can proceed via p38 kinase‐and caspase‐3‐dependent pathways that bypass classical ubiquitination. Additional non‐canonical routes, including lipid rafts or flotillins, have also been described [29]. Dysregulation of these processes can sustain EGFR signaling, promoting tumor progression and therapeutic resistance [30, 31].

TIMELESS‐interacting protein (TIPIN) is a well‐conserved factor initially characterized as a core component of the replication fork protection complex [32]. Canonically, TIPIN forms a heterodimer with TIMELESS to safeguard stalled replication forks, maintain genomic stability, and modulate DNA damage responses [33, 34, 35]. Mounting evidence indicates that elevated TIPIN expression exerts oncogenic effects across multiple human malignancies, facilitating tumor proliferation, survival, and genomic instability [36, 37]. However, nearly all existing studies focus on its nuclear functions related to DNA replication, and extra‐nuclear signaling roles of TIPIN remain largely unexplored. Whether TIPIN participates in regulating growth factor receptor turnover in liver cancer has not been investigated to date.

This work identifies TIPIN as an unrecognized regulator of EGFR stability in liver cancer. We show that TIPIN binds directly to EGFR and prevents c‐Cbl from ubiquitinating and degrading it in lysosomes, thereby maintaining EGFR signaling and enhancing oncogenic potential. Moreover, we show that TIPIN expression is epigenetically upregulated through promoter DNA hypomethylation, linking aberrant epigenetic regulation to sustained receptor activation. Functionally, TIPIN promotes lenvatinib resistance, whereas its depletion restores drug sensitivity. Collectively, these findings uncover a novel TIPIN–c‐Cbl–EGFR regulatory axis that drives malignant progression and therapeutic resistance in liver cancer.

## 2 Materials and Methods

### 2.1 Patient Samples

Liver cancer tissue specimens came from patients receiving surgical resection at the First Affiliated Hospital of Zhejiang University School of Medicine. Every case enrolled received histopathological confirmation. We excluded patients suffering from other severe comorbidities, active secondary malignant tumors, or individuals who had previously undergone radiotherapy, chemotherapy, or targeted‐agent therapy. Written informed consent was obtained from all study participants. Ethical approval for this work was granted by the Ethics Committee of the First Affiliated Hospital of Zhejiang University School of Medicine (Ethics Code: 2012–114; 2019‐1296‐1; 2021–384).

This study collected six independent patient cohorts. Cohort 1 included 12 pairs of tumor and adjacent specimens recruited from June 2015 to December 2019 to measure the abundance of TIPIN protein. Cohort 2 included 94 pairs of tumor and adjacent specimens obtained during the same period (from June 2015 to December 2019) for RNA‐seq analysis. Cohort 3 comprised 113 pairs of liver‐cancer specimens collected from 2012 to assess TIPIN and EGFR expression. Cohort 4 consisted of 9 pairs of tumor and adjacent specimens obtained starting from June 2015 for TIPIN protein detection. Cohort 5 included 10 pairs of tumor and matched adjacent non‐tumor samples obtained from April 2018 to January 2020 to analyze TIPIN's DNA methylation level. Cohort 6 enrolled 8 liver‐cancer tissues recruited from January 2019 to December 2024 to evaluate the correlation between TIPIN expression and lenvatinib treatment response. Complete clinical metadata of all patients are summarized in Tables S1–S6.

### 2.2 Construction of TIPIN Knockout Mice

TIPIN whole‐body knockout mice (*Tipin* ^−/−^) were generated by Shanghai Model Organisms Center, Inc. (Shanghai, China). On the C57BL/6J background, the CRISPR/Cas9 system was used to delete exon 3 and exon 4 of the canonical TIPIN transcript TIPIN‐205. Non‐homologous end joining takes place afterward and creates a frameshift mutation. Protein translation stops early because of this mutation, so no functional TIPIN protein can be made. The main steps to make these knockout mice are listed below. Researchers produced Cas9 mRNA and target‐specific gRNAs, which bind to intron 2 and intron 4 of the Tipin gene, using in vitro transcription. We combined Cas9 mRNA with the two matched gRNAs, then injected this mixture into fertilized C57BL/6J mouse zygotes to create F0 founder mice. Genotyping PCR and Sanger sequencing were performed to identify positive F0 founders, which were crossed with wild‐type C57BL/6J littermates to obtain stable heterozygous F1 progeny for subsequent breeding. The canonical transcript targeted in this TIPIN knockout line is TIPIN‐205 (ENSMUST00000216594.2).

### 2.3 Hydrodynamic Injection (HDI) Liver Tumor Model

Age‐matched male mice received hydrodynamic tail vein injection within 8 s. For every mouse, we delivered 2 mL of sterile PBS. This solution contained 10 µg Sleeping Beauty (SB) transposase plasmid, 25 µg C‐MYC plasmid, and 25 µg N‐RAS expression plasmid. After a consistent period of tumor growth and subsequent drug intervention, all mice were euthanized. We collected their entire livers to record liver weight and examine tumor phenotypes.

### 2.4 Cell Culture

Human liver cancer cell lines (Hep 3B, SNU449, PLC/PRF/5, Huh7 and SK‐Hep‐1), human umbilical vein endothelial cells (HUVECs), mouse liver cancer Hepa 1–6 cells, and HEK‐293T cells were all purchased from the Cell Bank of the Chinese Academy of Sciences (Shanghai, China). Cancer‐associated fibroblasts (CAFs, Cat# RY‐Y0072) were obtained from RuYao Biotechnology Co., Ltd. (Zhejiang, China), and their identity was validated by flow cytometry. All cells were grown in Dulbecco's Modified Eagle Medium (Thermo Fisher Scientific, MA, USA) supplemented with 10% fetal bovine serum (Gibco, MA, USA). Cells were kept in a humidified incubator at 37°C with 5% CO_2_ for stable growth. Mycoplasma contamination was checked using the MycAway Plus‐Color Mycoplasma Test Kit (YEASEN, Shanghai).

### 2.5 Transfection and Lentivirus Infection

For transient transfection, cells were plated into six‐well plates at 3 × 10^5^ cells per well. Anti‐TIPIN siRNAs were synthesized by Sunya Biological, while all plasmid constructs were generated by REPOBIO (Hangzhou, Zhejiang, China). Cells were transfected with JetPRIME transfection reagent (Polyplus 114‐01, Polyplus, Illkirch, France). Lentiviruses for TIPIN overexpression and short‐hairpin RNA (shRNA)‐mediated knockdown were supplied by Zorin Biological Company (Shanghai, China). Stable cell pools were selected with 5 µg/mL puromycin (Thermo Fisher Scientific, Waltham, MA, USA). Detailed sequences for the siRNAs and shRNAs can be found in Table S10.

### 2.6 RNA Sequencing and Data Processing

SK‐Hep‐1 cells were plated in a 6‐well plate and subjected to designated treatments for 24 h. Cells were harvested for total RNA extraction before RNA library construction and sequencing. RNA‐seq services were provided by Genedenovo Biotechnology Co., Ltd (Guangzhou, China) using the Illumina HiSeq 2500 platform. Raw sequencing reads were trimmed to remove adapters and low‐quality sequences with fastp. The resulting clean reads were aligned to the human GRCh38 reference genome using HISAT2. Gene expression quantification was performed via StringTie based on GENCODE gene annotation. Differential gene expression analysis was carried out using the DESeq2 package within the R programming environment.

### 2.7 EdU Assays

Cells were inoculated in 24‐well plates and incubated overnight. EdU assay was performed strictly following the instructions provided by the manufacturer (C6017, US EVERBRIGHT, USA). Subsequent to EdU labeling, the cells were subjected to fixation, permeabilization, and nuclear staining procedures.

### 2.8 Cell Co‐Culture Experiments

For co‐culture experiments with Hepa 1–6 cells and CAFs, 5 × 10^4^ liver cancer cells were plated into the lower chamber of 24‐well Transwell inserts, while 3 × 10^4^ CAFs were plated into the upper chamber. Separately, 3 × 10^5^ Liver cancer cells were cultured in 6‐cm dishes. The supernatants of the cell culture were harvested and centrifuged at 845 × g for 10 min to eliminate residual cell debris after 48 h. Collected supernatants were further sterilized using 0.22 µm microwell filter membranes. We mixed the prepared conditioned medium equally with fresh complete medium. This diluted medium was then used to culture CAFs or HUVECs for follow‐up assays.

### 2.9 Immunoblotting, Immunoprecipitation (IP), and Mass Spectrometry (MS)

For immunoblotting, cellular proteins were extracted using radioimmunoprecipitation assay (RIPA) buffer with 1% protease inhibitor cocktail added. Cell lysis was performed on ice for 30 min at 4°C. Total protein concentrations were quantified, and equal protein quantities were loaded and separated by SDS‐PAGE at 80 V and subsequently electrotransferred onto a polyvinylidene fluoride (PVDF) membrane (Millipore, Billerica, MA, USA). The membranes were blocked with standard western blocking buffer (Beyotime, Shanghai, China) for 1 h at room temperature. Afterward, the membrane was incubated overnight with the primary antibody in Table S11 at 4°C, and then incubated with the corresponding secondary antibody at room temperature for 1 h. Protein bands were visualized using the EZ‐ECL chemiluminescence detection kit (Thermo Fisher Scientific, Waltham, MA, USA). The band intensity was quantified using Fiji (ImageJ), normalized to the loading control.

Endogenous IP assays were conducted with the Dynabeads Co‐IP Kit (14321D, Thermo Fisher Scientific). For the detection of exogenous IP, magnetic beads pre‐conjugated with anti‐Flag or anti‐HA antibodies (B26102, B26202, BiMage, Houston, Texas, USA) were adopted for protein enrichment based on the manufacturer's operational guidelines. After thorough washing steps, the bound protein complex was eluted using 50 µL SDS loading buffer, and the eluted product was finally subjected to immunoblotting analysis.

### 2.10 Quantitative Real‐Time Polymerase Chain Reaction (qRT–PCR)

The RNA extraction kits purchased from Yishan Biotechnology Co. Ltd. (Shanghai, China) were utilized to isolate total cellular RNA. Purified RNA was then reverse transcribed into cDNA using the Vazyme reverse transcription kit (Nanjing, Jiangsu, China). Real‐time quantitative PCR was conducted on the QuantStudio 5 platform from Thermo Fisher Scientific. The ΔΔCt method was adopted to determine the relative mRNA expression profiles.

### 2.11 Colony Formation Assay

Six‐well plates were inoculated with cells at 2.5 × 10^3^ cells per well, followed by 2 weeks of incubation. Formed colonies were fixed and stained with 0.5% crystal violet (Beyotime, Shanghai, China) for subsequent colony counting.

### 2.12 Cell Migration and Invasion Assays

Transwell‐based experiments were employed to assess cellular migratory and invasive phenotypes using membranes from BD Biosciences. Migration tests utilized uncoated membranes, while Matrigel‐precoated membranes were adopted for invasion assays to mimic extracellular matrix (ECM) barriers. In total, 2 × 10^4^ cells suspended in 200 µL serum‑free medium were loaded into the upper insert. The lower compartment was filled with medium containing 10% fetal bovine serum to provide chemotactic stimulation. Cells were incubated for 3 days for migration detection and 5 days for invasion detection. Transmembrane cells were stained with 0.5% crystal violet and subjected to quantitative analysis.

### 2.13 Wound Healing Assay

Cells were incubated within Ibidi culture inserts (Martinsried, Germany) over 24 h to reach confluent growth. The inserts were then gently detached to generate uniform cell‐free gaps in the monolayer. Microscopic images were acquired (Zeiss, Oberkochen, Germany) at 0 and 24 h after gap formation.

### 2.14 Tumor Sphere Formation Assay

Single‐cell suspensions containing 1000 viable cells were prepared with serum‐free medium and dispensed into Corning ultra‐low‐adhesion 6‐well plates for sphere‐forming culture. After 10 days of incubation, tumor spheres were examined under a microscope; brightfield images were acquired for subsequent evaluation.

### 2.15 Trypsin Digestion Assay

Six‐well plates were inoculated with liver cancer cells at 8 × 10^5^ cells per well, followed by overnight incubation for cell adherence. The cells were either treated with 0.25% trypsin‐EDTA (Gibco) for 1–2 min or left untreated under standard incubation conditions (37°C). After removal of loosely digested cells, the adherent cell population was fixed with 4% paraformaldehyde (PFA) and stained with 0.5% crystal violet solution for subsequent phenotypic observation.

### 2.16 Adhesion Assay

Cell adhesive capacity was detected utilizing a commercial Cell Adhesion Kit (BestBio, Shanghai, China). First, 96‐well plates were treated with 100 µL coating solution per well and kept overnight at 4°C for sufficient coating. After incubation, the residual coating liquid was discarded, and all wells were rinsed three times with the matching washing solution included in the kit. Next, 1 × 10^5^ cells were seeded into each well and incubated for 30 min at 37°C in an incubator. At the end of incubation, the wells were washed three times to remove unadhered cells. Each well received Cell‑staining solution B prior to a 2‑h incubation maintained at 37°C. Quantification of cell adhesion was accomplished by detecting optical density values at 450 nm.

### 2.17 HUVEC Tube Formation Assay

Matrigel (356234; Corning Inc.) was applied onto pre‐chilled 96‐well plates, and plates underwent a 30 min incubation at 37°C for thorough gel polymer formation. HUVECs were resuspended in liver cancer cell‐derived conditioned medium at a density of 2 × 10^5^ cells/mL. A volume of 100 µL of this suspension was then seeded into each well of the 96‐well plate and incubated at 37°C for 6–18 h. After incubation, capillary‐like tube structures were observed under a microscope. The tube junction numbers were counted and statistically analyzed using ImageJ software.

### 2.18 Dual‐Luciferase Reporter System

Recombinant plasmids carrying wild‐type and mutant *TIPIN* promoter fragments were obtained from REPOBIO (Hangzhou, Zhejiang, China). For cell preparation, HEK‐293T cells (1 × 10^5^ cells per well) were cultured in 24‐well plates and allowed to adhere overnight before transfection. Co‐transfection was performed using the *TIPIN* promoter plasmid in combination with the pcDNA3.1‐DNMT1 plasmid (REPOBIO). For the mutant TIPIN promoter, co‐transfection included anti‐*DNMT1* siRNA (Table S10). A positive control was established by transfecting the pRL‐TK luciferase plasmid (REPOBIO). Forty‐eight hours following transfection, cellular luciferase signals were measured with the Dual Luciferase Reporter Assay Kit (Promega, Madison, WI, USA).

### 2.19 Immunofluorescence

Cells were plated at a density of 1 × 10^4^ cells in confocal culture dishes (NEST, Jiangsu, China) and fixed using 4% PFA. Samples were then blocked for 1 h using a phosphate‐buffered saline solution containing 0.5% Triton X‐100 and 4% bovine serum albumin. For target protein labeling, cells were incubated overnight at 4°C with specific primary antibodies, as listed in Table S11. This was followed by a 1‐h incubation with secondary antibodies (EarthOx, Millbrae, CA, USA). After washing three times, cell nuclei were stained with 4’,6‐diamidino‐2‐phenylindole (DAPI, Sigma–Aldrich, St. Louis, MO, USA) for 5 min. Fluorescence imaging was finally performed via a confocal microscope (Olympus, Tokyo, Japan).

### 2.20 Immunohistochemistry (IHC) and Masson's Trichrome Staining

Paraffin‐embedded tissue sections were processed for heat‐mediated antigen retrieval using citrate buffer (Beyotime, Shanghai, China). After dewaxing and gradient dehydration, samples were blocked with Beyotime blocking buffer for 30 min to reduce non‐specific binding. Sections were incubated with primary antibodies at 4°C overnight, then treated with secondary antibodies (ZSGB‐BIO, Beijing, China) for 30 min. Sections were then visualized using diaminobenzidine, counterstained, dehydrated, and mounted.

Staining strength was graded on a four‐point scale: 0 (negative), 1 (weak), 2 (moderate), and 3 (strong). Positive cell proportions were classified into five grades: 0 (0%), 1 (1%–25%), 2 (26%–50%), 3 (51%–75%), and 4 (> 75%). The composite immunohistochemistry score was derived by multiplying staining intensity values by the proportion of positive‐stained cells, with scores less than 4 considered low and scores of 4 or higher considered high. All staining results were independently assessed by two researchers to ensure accuracy.

Commercially supplied Masson's trichrome staining kit (Solarbio, Beijing, China) was utilized, and all experimental steps strictly complied with the kit's official instructions.

### 2.21 Bio‐Layer Interferometry (BLI) for Analysis of TIPIN‐EGFR Interaction and Competitive Binding

BLI experiments were conducted to analyze TIPIN‐EGFR interaction and competitive binding. Biotinylated EGFR (69.95 kDa) was prepared by equimolar biotinylation followed by overnight dialysis against 1×PBS, then immobilized on streptavidin sensors (Sartorius, 18–5019). His‐tagged TIPIN (39.3 kDa) was immobilized on NTA sensors (Sartorius, 18–5101). Both proteins were diluted to 5 µg/mL in SD buffer for 600s solidification. TIPIN, c‐Cbl, and CHEK1 were serially diluted in SD buffer to generate concentrations ranging from 1 down to 0.0625 µM. Assays included equilibration, binding, and dissociation phases with 96‐well black plates. Data were analyzed using Octet BLI Discovery software to determine kinetic parameters (OctetR8, Sartorius, Germany).

### 2.22 Apoptosis Analysis

Cell apoptosis was performed via Annexin V‐FITC and PI staining, following the standard protocols provided by Dojindo Molecular Technologies (Rockville, MD, USA). Cell apoptotic populations were examined using a BD flow cytometer (Becton, Dickinson and Company, Franklin, NJ, USA), and the corresponding apoptosis rates were quantitatively analyzed with FlowJo‐V10 software (BD Biosciences).

### 2.23 Reagents and Drugs Treatment

Liver cancer cells were seeded at 3 × 10^5^ cells in 6‐cm dishes. Cells were exposed to 5 µmol/L 5‐Azacytidine (AZA, MedChemExpress, Monmouth Junction, NJ, USA) for 24 h and harvested 48 h later for immunoblot analysis.

To investigate whether TIPIN knockdown‐mediated EGFR downregulation involves the proteasomal or lysosomal pathway, cells plated at 3 × 10^5^ cells per 6‐cm dish were exposed to 10 µmol/L MG132 or 20 µmol/L chloroquine (CQ, MedChemExpress, Monmouth Junction, NJ, USA).

TIPIN‐overexpressing (LV‐TIPIN) and control (LV‐Ctrl) liver cancer cells (Zorin Biological Company, Shanghai, China) were plated at 3 × 10^5^ cells per dish and exposed to 10 µmol/L cycloheximide (CHX, MedChemExpress, Monmouth Junction, NJ, USA). Cell lysates were gathered at 0, 2, 4, 8, 16, and 24 h following drug addition for immunoblotting. Meanwhile, shTIPIN and shCtrl cells (Table S10) cultured at the same density were treated with 5 µmol/L lenvatinib. Cell samples were harvested after 48 h for subsequent analysis.

### 2.24 DNA Methylation and Transcriptome Sequencing

Targeted‑bisulfite sequencing based on the Illumina MiSeq platform was utilized to profile DNA methylation signatures of clinical samples, and this service was accomplished by Genesky Biotechnology Inc. (Shanghai, China). Bisulfite sequencing PCR assays were applied to examine methylation levels in liver cancer cell lines, and the technical service was provided by Cosmos Wisdom Biotechnology Co., Ltd. (Hangzhou, Zhejiang, China). Transcriptome sequencing was performed on tissues from cohort 2. In parallel, TIPIN‑knockdown SK‑Hep‑1 cells and control counterparts were harvested for RNA‑seq library construction and sequencing on the Illumina HiSeq 2500 system by Genedenovo Co. Ltd. The DESeq2 R package was used to screen differentially expressed genes, whereas Gene Ontology functional enrichment was analyzed with the online Omicsmart platform.

### 2.25 Public Database Analysis

We leveraged two open‑access databases, TCGA (http://www.cbioportal.org) and GEO (https://www.ncbi.nlm.nih.gov/geo), for functional validation of TIPIN in liver cancer. CpG‑island patterns in the TIPIN promoter sequence were predicted using the EMBOSS tool (https://www.ebi.ac.uk/Tools/seqstats/emboss_cpgplot). Protein‐protein interaction networks were predicted via the STRING database (https://string‐db.org). Additionally, potential EGFR‐targeting E3 ubiquitin ligase candidates were screened on the UbiBrowser portal (http://ubibrowser.bio‐it.cn/ubibrowser_v3).

### 2.26 Animal Experiments

All experimental mice were purchased from the Shanghai Experimental Animal Center, Chinese Academy of Sciences. Animals were raised in a specific pathogen‐free (SPF) environment under standardized conditions: a 12‐h light‐dark cycle, constant temperature of 24°C, and 50% ambient humidity. Mice were euthanized via carbon dioxide asphyxiation upon experiment completion. Animal experimental protocols were approved by the Animal Care Committee of Zhejiang University (Ethics Code: 2019‐1218) and conducted in strict compliance with the NIH Guide for the Care and Use of Laboratory Animals.

For the subcutaneous xenograft model, 4–6‐week‐old male BALB/c nude mice were randomly assigned to four experimental groups: sh‐Ctrl, sh‐TIPIN, lenvatinib, and sh‐TIPIN combined with lenvatinib. Mice received subcutaneous injection of liver cancer cells, and drug intervention was initiated one week after cell inoculation. Mice were orally administered lenvatinib (30 mg/kg/day, 1.2 mg/mL dissolved in 5% DMSO) or 5% DMSO vehicle saline for four consecutive weeks. Tumor tissues were dissected after mouse euthanasia and weighed for subsequent statistical analysis.

To establish the lung colonization metastasis model, each mouse received tail vein injection of 2 × 10^6^ liver cancer cells. Mice were euthanized two months after cell inoculation. Isolated lung tissues were fixed in 4% PFA and stained with hematoxylin and eosin (H&E). Metastatic nodules across entire lung lobes were quantified under microscopy.

### 2.27 PDO and PDX Models

Primary liver cancer cells isolated from clinical specimens were embedded within suspended hydrogels to generate PDOs. The hydrogel matrix consisted of 0.5% alginate mixed with 0.25% gelatin (Sigma–Aldrich, St. Louis, MO, USA), and crosslinking was achieved using 75 mmol/L CaCl_2_ solution (Solarbio, Beijing, China). Cells and cell clusters were homogeneously mixed with hydrogel before crosslinking. After solidification, PDOs were stabilized by 5 days of routine culture prior to drug intervention and maintained for another 7 days afterward. Calcein‐AM/PI double staining kit was used to detect cell viability (Dojindo Molecular Technologies). All PDX operations were approved by the Ethics Committee of the First Affiliated Hospital of Zhejiang University School of Medicine. Fresh clinical tumor tissues were rinsed with ice‐cold DMEM, trimmed into 1–2 mm^3^ tissue fragments, and implanted subcutaneously on the back of NSG mice.

### 2.28 Statistical Analysis

GraphPad Prism 9.0 (GraphPad, San Diego, CA, USA) was used to conduct statistical evaluation. Quantitative results were expressed as mean ± SD. Paired or unpaired Student's t‐tests were employed to evaluate differences between two groups. Pearson correlation analysis was applied to evaluate variable relationships. All findings were derived from at least three independent experiments. Statistical significance was set at ^*^ *p* < 0.05, ^**^ *p* < 0.01, ^***^ *p* < 0.001, and ^****^ *p* < 0.0001.

## 3 Results

### 3.1 Genome‐Wide Methylation Profiling Identifies TIPIN as the Most Significantly Altered Gene in Liver Cancer

DNA methylation is a key epigenetic modification frequently associated with gene silencing in various cancers, including liver cancer. Changes in DNA methylation patterns play a significant role in tumor progression and therapy resistance. Given the critical role of DNA methylation in regulating gene expression, we aimed to identify genes with significantly altered methylation patterns in liver cancer. To screen for such genes, we analyzed TCGA‐LIHC DNA methylation data (Table S7) with the following criteria: (1) DNA methylation sites located in the promoter region, (2) |log_2_FC| > 0.25 (FC: fold change of cancer vs normal control), (3) FDR < 0.001 (FDR: false discovery rate), and (4) *p* < 0.05 for correlation between gene expression levels and overall survival (OS) or disease‐free survival (DFS). Patients were split into high‐ and low‐expression subgroups according to the median mRNA value of each gene for survival analysis. The log‐rank test was used to compare OS and DFS between groups. Note that this was a univariate survival analysis without adjustment for additional clinical covariates. We further filtered CpG probes to retain those displaying a statistically significant negative correlation between probe methylation level and corresponding gene mRNA expression (*p* < 0.05). Similarly, TCGA RNA‐seq data were screened with the following criteria: (1) the log_2_FC of RNA sequencing shows the opposite trend to DNA methylation levels, (2) FDR < 0.05, and (3) *p* < 0.05 for OS or DFS. From this integrated analysis, the top ten (PTTG1, SFN, PRMT3, ANXA2, AP1S1, TUBA1B, ENO1, TIPIN, SERPINE2, HDAC7) hits were identified (Table S8), of which three (AP1S1, TIPIN, HDAC7) have not been previously reported in liver cancer. To further prioritize candidates, we performed targeted‐promoter methylation sequencing using paired tumor‐adjacent non‐tumor tissues from our liver cancer clinical cohort (Table S9). Of the three genes, TIPIN showed the greatest disparity in promoter methylation between tumor and paired adjacent non‐tumor tissues; we thus prioritized TIPIN for further functional validation (Figure 1A).

**FIGURE 1:**
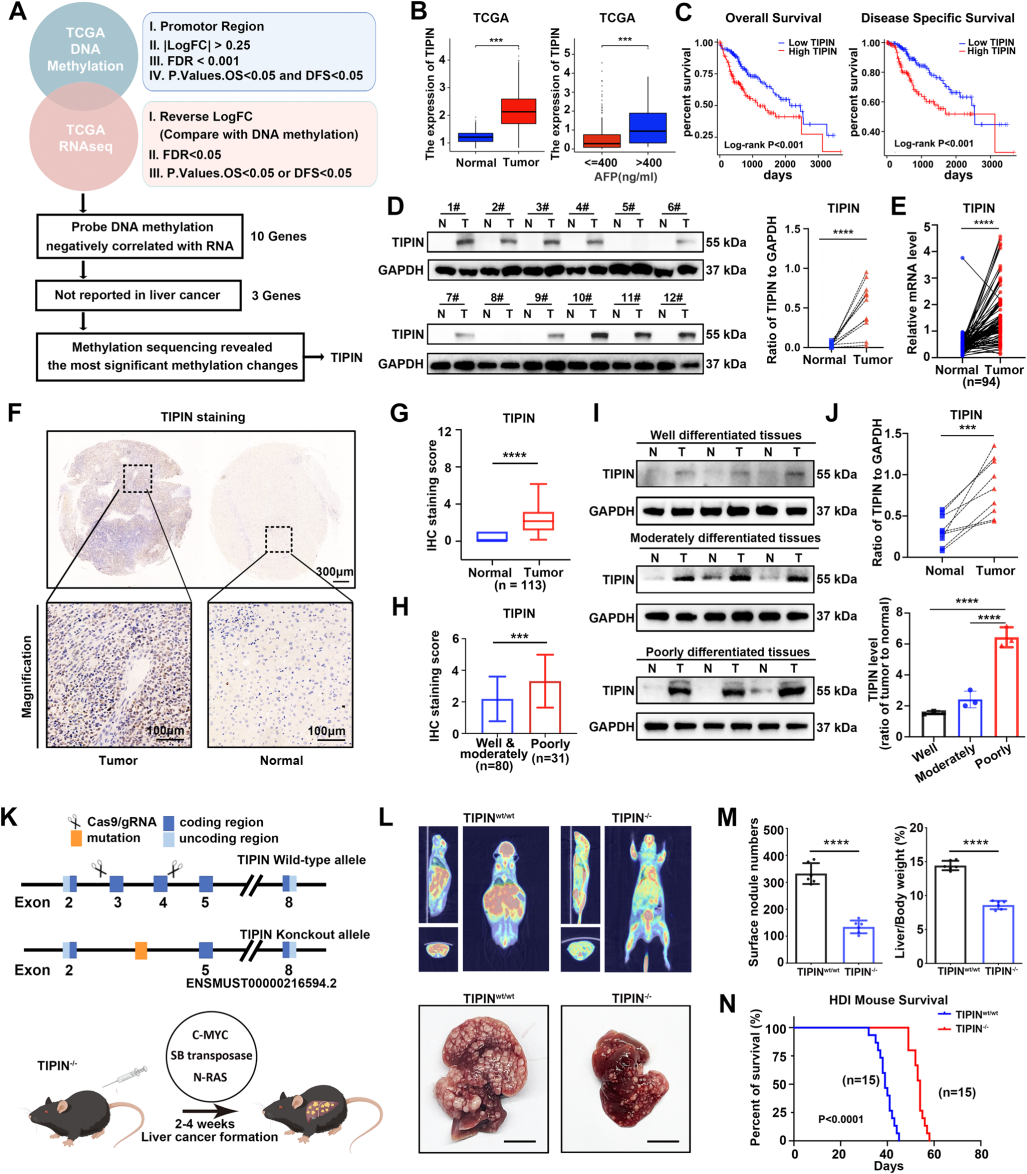
High expression of TIPIN is closely related to the progression of liver cancer. (A) Schematic workflow of TIPIN identification via the TCGA dataset combined with literature review. TCGA DNA methylation screening criteria: (1) Methylation sites mapped to gene promoter regions; (2) |Log_2_ FC| > 0.25; (3) FDR < 0.001; (4) *p* < 0.05 for OS and p < 0.05 for DFS. TCGA RNA‐seq screening criteria: (1) Log_2_FC of RNA sequencing showing the opposite trend to DNA methylation; (2) FDR < 0.05; (3) for either OS or DFS. (B) TCGA dataset analysis revealed elevated TIPIN expression in tumor specimens relative to normal tissues (left, mean ± SD, unpaired Student's t‐test). TIPIN expression exhibits a positive correlation with AFP levels within TCGA (right, mean ± SD, unpaired Student's t‐test; only tumor samples were included in this analysis). (C) Survival curves derived from the TCGA cohort illustrate that elevated TIPIN expression confers inferior prognosis in liver cancer patients (Log‐rank test). (D) Immunoblot analysis was performed to assess TIPIN protein levels in tumor tissues and the adjacent non‐tumor counterparts (Cohort 1, *n* = 12). The right panel displays the relative quantitative results of the immunoblots (means ± SD, paired Student's t‐test). (E) RNA sequencing data demonstrated that TIPIN expression was markedly higher in liver tumor tissues than in their matched adjacent non‐tumor counterparts of Cohort 2. (F) Representative tissue microarray (TMA) staining images showing TIPIN levels in liver tumor and matched adjacent non‐tumor tissues of liver cancer patients (Cohort 3, *n* = 113). (G) TIPIN IHC scores in liver tumor and matched adjacent non‐tumor tissues (Cohort 3, *n* = 113). (H) TIPIN IHC scores across different tumor differentiation grades (Cohort 3, *n* = 111; 2 samples were excluded due to incomplete differentiation records). (I) Immunoblot was used to detect TIPIN expression in liver cancer and paired adjacent non‐tumor tissues of different degrees of differentiation in Cohort 4. (J) Densitometric quantification of TIPIN expression in liver tumor and matched adjacent non‐tumor tissues based on immunoblot assays (up). Comparison of immunoblot quantification results between well‐differentiated and poorly‐differentiated tumor specimens (down, mean ± SD, unpaired Student's t‐test). (K) Generation of TIPIN knockout (*TIPIN* ^−^ *^/^* ^−^) mice and establishment of HDI model. *TIPIN* ^−/^ *^−^* mice were generated via CRISPR/Cas9‑mediated precise deletion of the coding regions within the TIPIN transcript (ENSMUSG00000032397). For HDI model induction, 6‐week *TIPIN* ^−^ *^/−^* mice and wild‐type (*TIPIN^wt/wt^*) mice received a rapid tail vein injection of 2 mL PBS supplemented with 10 µg Sleeping Beauty (SB) transposon, 25 µg C‐MYC plasmid, and 25 µg N‐Ras plasmid. All tumor‐related analyses shown in figures 1L–N were performed using this HDI‐driven tumor model. (L) PET/CT results and representative liver images of mice 4 weeks after HDI model establishment (scale bars, 1 cm). (M) Quantitative analysis of surface nodule numbers and liver/body weight ratio in the HDI liver cancer model (*n* = 5 per group, mean ± SD, unpaired Student's t‐test). (N) Kaplan‐Meier survival comparison between *TIPIN^wt/wt^* mice and *TIPIN* ^−^ *^/^* ^−^ mice in the HDI tumor model (*p* < 0.0001, Log‐rank test).

As a hypomethylated gene, TIPIN expression was upregulated in liver cancer and correlated with alpha‐fetoprotein (AFP) levels (Figure 1B). Higher TIPIN expression was associated with poorer survival outcomes (Figure 1C). Immunoblotting analysis confirmed increased TIPIN protein levels in liver cancer tissues (Cohort 1, *n* = 12, Figure 1D). RNA‐seq analysis of 94 paired liver tumor and non‐tumor specimens (Cohort 2) showed elevated TIPIN levels in malignant tissues (Figure 1E), a result consistent with TCGA‐LIHC data. IHC on tissue microarrays of Cohort 3 further demonstrated significantly increased TIPIN expression in the tumor tissues (Figure 1F,G). Moreover, TIPIN IHC scores inversely correlated with tissue differentiation status (Figure 1H). Immunoblotting analysis of liver cancer with different differentiation status and adjacent tissues (Cohort 4, *n* = 9) validated that higher levels of TIPIN were observed in more poorly differentiated tissues (Figure 1I,J).

To explore the functional involvement of TIPIN in liver cancer progression, whole‐body TIPIN knockout (*TIPIN* ^−^ *^/^* ^−^) mice were generated. We first examined naive adult TIPIN wild‐type (*TIPIN^wt/wt^*) and *TIPIN* ^−^ *^/^* ^−^ mice. Gross liver morphology, H&E staining, and serum ALT/AST assays revealed no spontaneous liver injury or histological defects in *TIPIN* ^−^ *^/^* ^−^ mice (Figure S1A,B). The *TIPIN^wt/wt^* and *TIPIN* ^−^ *^/^* ^−^ mice were used to establish HDI models for primary liver tumorigenesis over 2–4 weeks (Figure 1K). PET/CT imaging and subsequent tissue analysis revealed that *TIPIN^wt/wt^* mice developed significantly more surface tumor nodules and had an elevated liver weight–to–body weight (LW/BW) ratio compared with *TIPIN* ^−^ *^/−^* mice (Figure 1L,M). Consistently, *TIPIN^wt/wt^* mice exhibited worse survival outcomes (Figure 1N).

### 3.2 Hypomethylation of TIPIN Leads to Elevated Expression in Liver Cancer

Analysis of the TCGA database revealed significant hypomethylation of TIPIN in liver cancer tissues (Figure S1A). Six hypomethylated CpG sites were identified, each showing a strong negative correlation between methylation levels and TIPIN expression, exemplified by probe cg11164506 (Figure 2A; Figure S1B–D). Patients with TIPIN hypomethylation exhibited a poorer prognosis (Figure 2B). Consistently, GEO datasets also confirmed reduced TIPIN methylation in liver cancer tissues (Figure 2C). EMBOSS analysis predicted a CpG island (UR_CpG) in the *TIPIN* promoter that had not been documented previously (Figure S2A; Figure 2D). Methyl‐target sequencing of ten matched tumor‐normal pairs (Cohort 5) further validated TIPIN hypomethylation in tumors (Figure 2E,F; Figure S2B).

**FIGURE 2:**
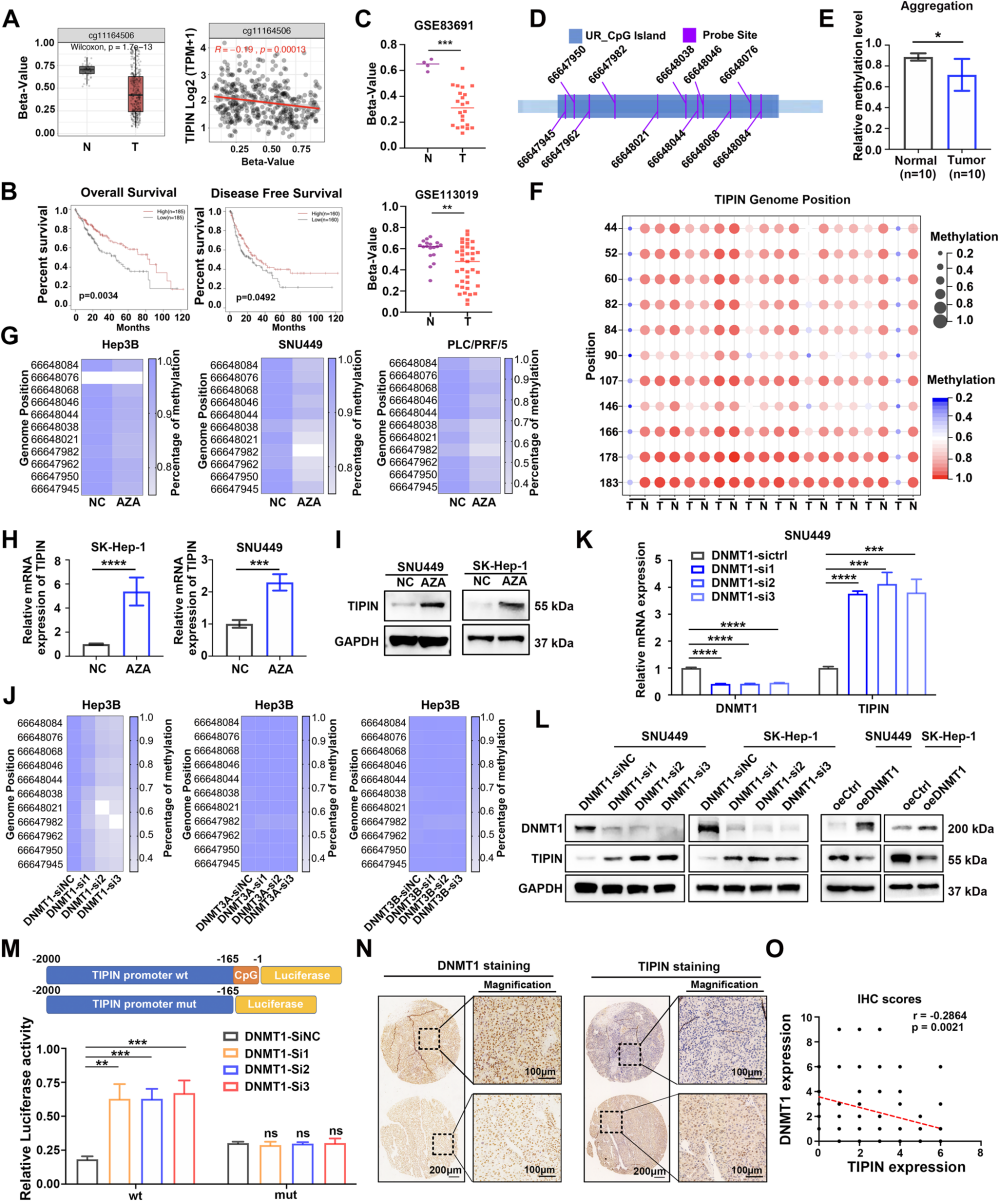
DNA methylation modulates TIPIN expression, and TIPIN levels are inversely correlated with patient prognosis. (A) Relative methylation level of TIPIN probes (cg11164506) in liver cancer specimens within the TCGA dataset (left). The Pearson correlation between TIPIN methylation and its mRNA expression in TCGA databases (right). (B) Kaplan‐Meier survival analysis was conducted with patients stratified into high and low TIPIN methylation groups as determined by probe cg1164506. (C) Methylation status of probe cg11164506 in GSE83691 and GSE113019 datasets (mean ± SD, unpaired Student's t‐test). (D) Schematic illustration of 10 methylation probes designed for 11 sites in the TIPIN unreported CpG (UR_CpG) island. (E) Site‐specific DNA methylation quantification across 11 probe sites in the TIPIN UR_CpG island, using 10 paired tissue samples from Cohort 5 (mean ± SD, paired Student's t‐test). (F) Shown is a heatmap derived from targeted methylation sequencing, depicting the methylation status of 13 CpG loci within the target CpG islands in the TIPIN promoter region. The assay was conducted on 10 matched tumor‐adjacent normal tissue pairs from our center. T: tumor tissue; N: normal tissue. Horizontal axis: probe positions (numbers = base locations on the CpG island, e.g. 44 = the 44th base site). Dot size and red intensity are positively associated with methylation level. (G) Targeted methylation sequencing was carried out to characterize the methylation state of the UR_CpG island in liver cancer cells with or without AZA treatment. (H, I) RT‐qPCR and immunoblot assays were applied to quantify TIPIN transcript and protein abundance, respectively, in liver cancer cells with or without AZA intervention (mRNA data are shown as mean ± SD, unpaired Student's t‐test). (J) DNA methylation profiling was conducted in Hep3B cells knocking down DNMTs (DNMT1, DNMT3A or DNMT3B). (K) RT‐qPCR detection of TIPIN expression upon DNMT1 knockdown. (L) Immunoblot analysis of TIPIN expression upon DNMT1 knockdown (left) and ectopic DNMT1 overexpression (right) in liver cancer cells. (M) Luciferase reporter plasmids carrying the wild‐type TIPIN promoter and its deletion mutant were constructed, respectively. Relative luciferase activity of the two promoter constructs was detected in SK‐Hep‐1 cells upon DNMT1 knockdown. (N) Representative TMA‐stained images showing TIPIN and DNMT1 levels in tumor tissues of liver cancer patients (Cohort 3). (O) The correlation between TIPIN and EGFR protein levels was analyzed via IHC (Pearson's correlation test).

In liver cancer cells, the DNA methyltransferase inhibitor azacitidine lowered TIPIN methylation while raising its transcript levels (Figure 2G–I; Figure S2C). Among the DNA methyltransferases examined, only DNMT1 knockdown significantly reduced TIPIN methylation (Figure 2J) and increased TIPIN expression (Figure 2K,L); Consistently, ectopic overexpression of DNMT1 suppressed endogenous TIPIN protein levels (Figure 2L; Figure S2D). Dual‐luciferase reporter assays confirmed that TIPIN promoter activity was suppressed by DNMT1 (Figure 2M). IHC staining of Cohort 3 tissues demonstrated a notable negative correlation between the protein expression levels of TIPIN and DNMT1 (Figure 2N,O). Collectively, these results demonstrate that TIPIN expression is wired to DNA‐methylation‐based epigenetic control, with DNMT1 acting as a key mediator of its transcriptional repression.

### 3.3 TIPIN Promotes Growth and Metastasis of Liver Cancer

To clarify how TIPIN contributes to liver cancer, we first evaluated its effects on tumor cell proliferation. Huh7 and SK‐Hep‐1 cells show relatively abundant endogenous TIPIN expression (Figure S2E), so we chose these two lines for subsequent functional experiments. This selection allows us to capture clear phenotypic changes and reliably describe the biological effects of TIPIN loss. TIPIN knockdown markedly reduced cell growth, colony formation, EdU incorporation, and tumor sphere formation efficiency (Figure 3A–E; Figure S3A). Subcutaneous and orthotopic xenograft models further demonstrated that TIPIN depletion significantly impaired tumor growth in vivo (Figure 3F,G).

**FIGURE 3:**
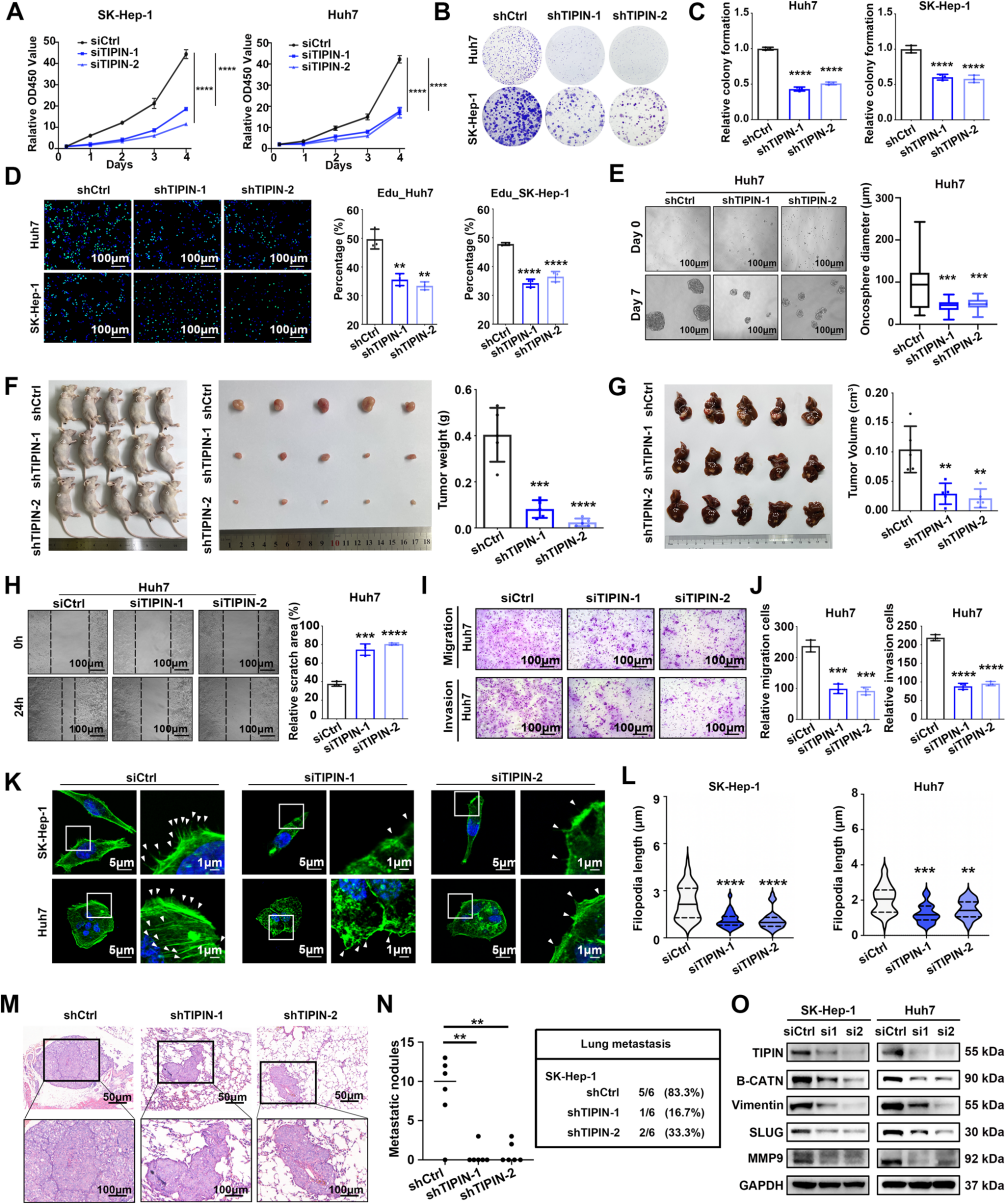
TIPIN knockdown inhibited the proliferation, cell adhesion, migration, and invasion of liver cancer cells. (A) Proliferative capacity of Huh7 and SK‐Hep‐1 cells transfected with TIPIN siRNA or siCtrl was evaluated via the CCK‐8 assay. (B, C) Colony assays of cells infected with TIPIN siRNA or siCtrl (mean ± SD, unpaired Student's t‐test). (D) Cell proliferation was assessed by EdU incorporation assay in Huh7 and SK‐Hep‐1 cells with TIPIN siRNA or siCtrl treatment (mean ± SD, unpaired Student's t‐test). (E) The oncosphere formation ability of liver cancer cells was compared between shTIPIN and shCtrl groups (mean ± SD, unpaired Student's t‐test). (F) Subcutaneous tumors of liver cancer cells in shTIPIN and shCtrl groups (*n* = 5 per group) and the tumor weight (g) were quantified for each group (mean ± SD, unpaired Student's t‐test). (G) Orthotopic liver tumor models were generated using shTIPIN and shCtrl cells (*n* = 5 per group). Representative images are presented, with tumor regions highlighted by white circles. (H) Wound‐healing assays were performed to evaluate the migratory motility of Huh7 cells in the indicated groups at 0 and 24 h (*n* = 3 each group, mean ± SD, unpaired Student's t‐test). (I, J) Transwell migration and invasion assays were performed in Huh7 cells transfected with TIPIN siRNA or siCtrl. Representative images and corresponding quantification are shown (*n* = 3 each group, mean ± SD, unpaired Student's t‐test). Scale bars, 100 µm. (K, L) Immunofluorescence visualization of the cytoskeleton in treated cells: F‐actin stained with phalloidin (green), nuclei counterstained with DAPI (blue), pseudopodia marked by white arrowheads. Scale bars: 5 or 2 µm. (L) Violin plots showing quantitative measurement of filopodia length. Filopodia length was manually traced and measured on reconstructed confocal images using Fiji (ImageJ). Lamellipodia structures were excluded from quantification. (M, N) H&E representative micrographs illustrate lung metastatic lesions in shCtrl and shTIPIN groups. Comparative analysis of pulmonary metastasis incidence across the shCtrl and shTIPIN experimental cohorts. (O) Immunoblotting results of the indicated proteins in Huh7 and SK‐Hep‐1 cells transfected with TIPIN siRNA or siCtrl.

Results from Transwell and wound healing experiments indicated that TIPIN suppression impaired cell migration and invasion (Figure 3H–J; Figure S3B,C). Cell adhesion assays also showed lowered adhesive properties after TIPIN knockdown (Figure S3D,E). Conversely, TIPIN overexpression strengthened cell adhesion and motility (Figure S3F–I). Immunofluorescence staining demonstrated fewer pseudopodia in TIPIN‐suppressed cells (Figure 3K,L), in line with transcriptomic profiling results (Figure S3J–O). TIPIN knockdown significantly decreased both the number and size of metastatic nodules (Figure 3M) and reduced the overall metastasis rate (Figure 3N). Mechanistically, TIPIN suppression downregulated multiple epithelial–mesenchymal transition (EMT)–related proteins (Figure 3O; Figure S3P). Taken together, these findings demonstrate that TIPIN enhances liver cancer proliferation, migration, invasion, and metastatic progression.

### 3.4 TIPIN Directly Interacts With EGFR

IP‐MS analysis was performed in Huh7 and SK‐Hep‐1 cells (Figure 4A), in combination with the BioGrid database (https://thebiogrid.org/), to identify potential TIPIN‐interacting proteins (Figure 4B). Notably, EGFR emerged as an especially interesting interactor given its well‐established roles in tumor progression and drug resistance, as well as strong translational relevance in liver cancer. Importantly, the physical interaction between TIPIN and EGFR has not been documented in prior studies. Representative MS/MS spectra show identified peptide fragments of endogenous EGFR and TIPIN obtained from immunoprecipitation samples of SK‐Hep‐1 and Huh7 cells (Figure 4C,D). Molecular docking (https://zdock.wenglab.org) further predicted a direct TIPIN–EGFR interaction (Figure 4E). In liver cancer cells, immunofluorescence staining showed partial cytoplasmic co‐localization between TIPIN and EGFR in SK‐Hep‐1 and Huh7 cells, supported by fluorescence line‐scan intensity profiles (Figure 4F), and the endogenous Co‐IP assays confirmed their physical interaction (Figure 4G). Exogenous Co‐IP assays further validated this interaction (Figure 4H). To map the specific binding regions, we constructed a series of EGFR and TIPIN truncation mutants. Co‐IP analysis showed that EGFR interacts with TIPIN through its intracellular domain (ICD) (Figure 4I). Among TIPIN truncations, TIPIN‐Flag‐Δ1‐143 showed the weakest binding affinity to EGFR (Figure 4J), and immunoblotting revealed that this mutant failed to promote EGFR expression (Figure 4K). Finally, BLI assays confirmed a concentration‐dependent increase in TIPIN–EGFR binding affinity (Figure 4L).

**FIGURE 4:**
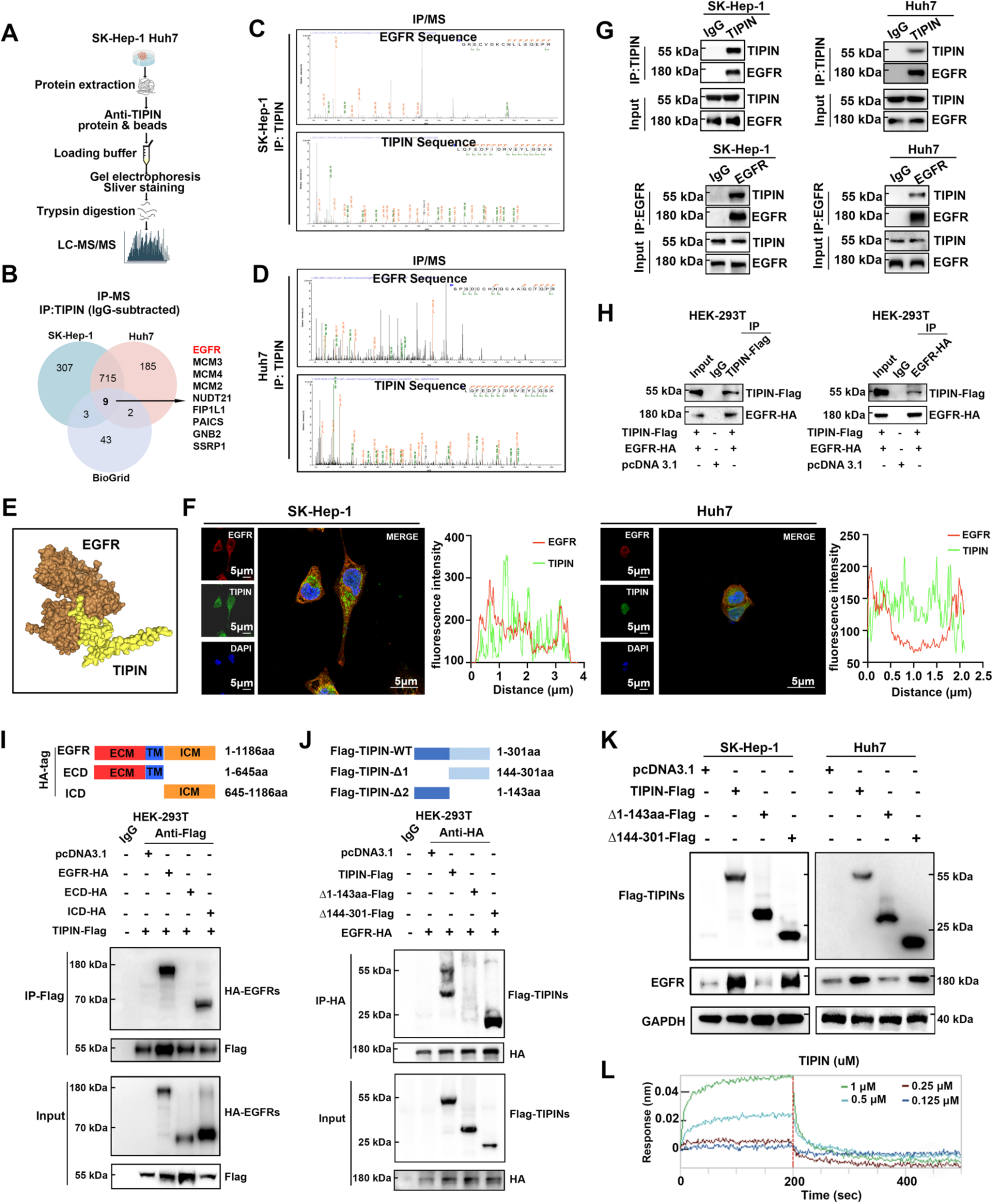
TIPIN was demonstrated to interact with EGFR. (A–D) IP/MS workflow schematic for the identification of EGFR as a TIPIN‐binding partner (A); Venn diagram showing shared candidate interactors from TIPIN IP‐MS (IgG subtracted) in two cell lines. Nine overlapping hits intersecting with BioGrid‐predicted TIPIN interactors are indicated, including EGFR (B); Secondary mass spectrum showing an EGFR‐derived peptide (C, D). (E) Molecular docking predicted the interaction between TIPIN and EGFR. (F) Immunofluorescence assay for the co‐localization of TIPIN and EGFR (scale bars, 5 µm). Representative confocal immunofluorescence image illustrating the subcellular distribution of EGFR (red) and TIPIN (green) in SK‐Hep‐1 cells. The line‐scan fluorescence intensity profile along the marked path demonstrates partially overlapping signal peaks for EGFR and TIPIN, indicating cytoplasmic co‐localization. (G) Endogenous binding detected in liver cancer cells. (H) Exogenous binding assay in HEK‐293T cells overexpressing Flag‐tagged TIPIN and HA‐tagged EGFR. (I, J) Truncation mutants of TIPIN and EGFR were constructed based on UniProt domain annotations. Interactions between the respective truncations were assessed by IP in HEK‐293T cells. (K) Immunoblot analysis of EGFR protein levels in liver cancer cells transfected with different TIPIN truncations. (L) Protein‐protein affinity assay for TIPIN‐EGFR binding.

### 3.5 TIPIN Regulates EGFR Signaling in Liver Cancer Cells

IHC evaluation identified a clear positive relationship between TIPIN and EGFR levels in liver cancer tissues (Figure 5A,B). Transcriptome sequencing further indicated that the ERK1/2 signaling pathway was markedly downregulated in siTIPIN cells when compared with siCtrl controls (Figure 5C). Consistent with this, immunoblotting confirmed that TIPIN knockdown reduced EGFR levels as well as its downstream STAT3 and ERK signaling (Figure 5D; Figure S4A). In contrast, TIPIN overexpression in Huh7 and SK‐Hep‐1 cells resulted in profound activation of EGFR signaling, as evidenced by the enhanced phosphorylation of EGFR, STAT3, and ERK (Figure 4B).

**FIGURE 5:**
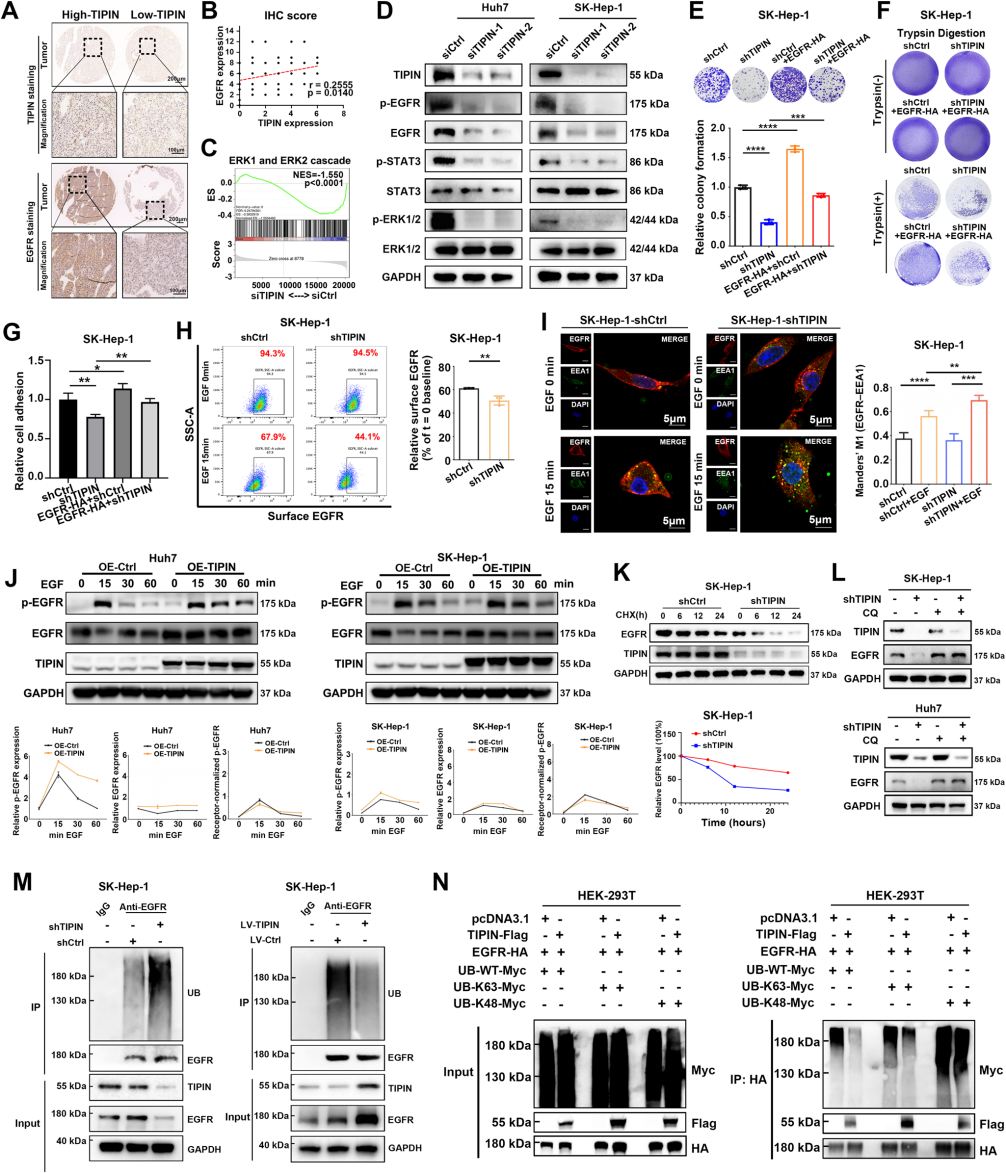
TIPIN stabilizes EGFR through inhibition of the lysosome‐mediated proteolytic pathway. (A) Representative IHC images showing co‐expression pattern of TIPIN and EGFR in liver cancer tissue specimens. (B) Pearson's correlation analysis was conducted to assess the expression association between TIPIN and EGFR based on IHC staining results. (C) GSEA results of ERK1/2 related pathway in the RNA‐seq data from liver cancer cells under the indicated treatments. (D) Immunoblot analysis of EGFR and its downstream signaling proteins in TIPIN‐knockdown liver cancer cells. (E–G) Ectopic EGFR expression rescues the inhibitory effects of TIPIN knockdown on cell proliferation (E) and adhesion (F, G) in liver cancer cells (mean ± SD, unpaired Student's t‐test). (H) Flow cytometric analysis and quantification of residual surface EGFR after EGF stimulation. Surface EGFR geometric mean fluorescence intensity (Geo‐MFI) at 15 min post‐EGF was normalized to t = 0 baseline within each group (mean ± SD, unpaired Student's t‐test). (I) Immunofluorescence analysis of EGFR surface levels on liver cancer cells after EGF stimulation. Manders’ M1 coefficient was calculated to quantify the proportion of EGFR overlapping EEA1‐positive early endosomes. Data are mean ± SD. ^**^ *p* < 0.01, ^***^ *p* < 0.001, ^****^ *p* < 0.0001. (J) Immunoblot analysis of p‐EGFR, total EGFR, and TIPIN in TIPIN‐overexpressing Huh7 and SK‐Hep‐1 liver cancer cells upon EGF stimulation at the indicated time points. Quantifications show relative p‐EGFR normalized to GAPDH, relative total EGFR normalized to GAPDH, and receptor‐normalized p‐EGFR (p‐EGFR/total‐EGFR). Data represent mean ± SD from the independent biological replicates. (K) CHX (10 µg/mL) was administered to lentivirally transduced SK‐Hep‐1 cells across the designated time points. The corresponding quantification of EGFR protein stability is presented. (L) EGFR protein levels in TIPIN‐knockdown liver cancer cells following CQ intervention or vehicle control treatment were examined via immunoblot analysis. (M) Evaluation of EGFR ubiquitination levels in liver cancer cells with TIPIN knockdown or overexpression. (N) EGFR ubiquitination with different ubiquitin mutants upon TIPIN expression. HEK‐293T cells were transiently co‐transfected with HA‐labeled EGFR and Myc‐labeled ubiquitin expression plasmids (WT, K63, K48), in the presence or absence of Flag‐labeled TIPIN. EGFR ubiquitination was assessed by immunoblotting.

To assess the functional impact of TIPIN on EGFR‐mediated oncogenic functions, we examined cell proliferation, adhesion, and metastasis. EGFR overexpression significantly rescued the inhibition of these processes caused by TIPIN knockdown (Figure 5E–G; Figure S4C–F). We further mapped the functional domain of TIPIN via truncated‐mutant rescue assays in TIPIN‐silenced SK‐Hep‐1 cells (Figure S4G–I). The N‐terminal fragment (1‐143 aa, Δ2), but not the C‐terminal fragment (144‐301 aa, Δ1), restored EGFR abundance, ERK1/2 activation, colony‐formation, and cell‐adhesion ability.

We next investigated the impact of TIPIN on EGFR internalization by measuring surface EGFR levels using flow cytometry. TIPIN knockdown induced a substantial reduction in cell‐surface EGFR abundance at 15 min after epidermal growth factor (EGF) stimulation (Figure 5H; Figure S5A). Additionally, after EGF stimulation, TIPIN‐knockdown cells exhibited a higher percentage of EGFR colocalized with the early endosome marker EEA1 compared to control cells, suggesting that TIPIN regulates EGFR internalization (Figure 5I). Upon EGF stimulation in Huh7 and SK‐Hep‐1 cells, TIPIN overexpression prolonged the duration of EGFR phosphorylation, whereas TIPIN knockdown accelerated the decay of EGFR‐dependent signaling (Figure 5J; Figure S5B). When p‐EGFR signals were normalized to total EGFR protein, phosphorylation kinetics were comparable between control and TIPIN‐manipulated groups in both cell lines. These results demonstrate that sustained EGFR phosphorylation upon TIPIN overexpression arises from stabilization of total EGFR protein, rather than enhancement of the intrinsic phosphorylation efficiency of individual EGFR receptors. We also assessed EGFR protein stability and observed that TIPIN knockdown markedly reduced EGFR half‐life (Figure 5K and Figure S6A). Interestingly, the lysosome inhibitor chloroquine rescued the EGFR degradation caused by TIPIN knockdown, while MG132 had no effect (Figure 5L; Figure S6B,C).

Given that EGFR stability relies heavily on ubiquitination [25], we explored whether TIPIN could modify the ubiquitination status of EGFR. TIPIN knockdown significantly enhanced EGFR ubiquitination, and TIPIN overexpression attenuated EGFR ubiquitination and degradation (Figure 5M; Figure S6D,E). Ubiquitination assays also revealed that TIPIN overexpression specifically decreased K63‐linked ubiquitination on EGFR (Figure 5N), suggesting that TIPIN regulates EGFR via the ubiquitination pathway.

### 3.6 TIPIN Displaces c‐Cbl From EGFR and Suppresses Ubiquitination‐Mediated EGFR Degradation

Ubiquitination is a key post‐translational modification that tags proteins with ubiquitin molecules to regulate their degradation or functional changes [38]. E3 ubiquitin ligases serve as the core enzymes that identify target substrates and drive the ubiquitination reaction [39]. To identify potential E3 ubiquitin ligases involved in EGFR ubiquitination, we searched the UbiBrowser database, which suggested c‐Cbl as a candidate (Figure S7A). STRING‐based bioinformatic screening singled out c‐Cbl as the E3 ubiquitin ligase within the top‐ten candidates predicted to interact with EGFR (Figure S7B). Consistent with its canonical role as an E3 ubiquitin ligase for EGFR, c‐Cbl significantly increased EGFR ubiquitination levels (Figure S7C). Similar to TIPIN, c‐Cbl binds to the EGFR's ICD (Figure 6A). We therefore generated truncation constructs targeting the EGFR ICD (Figure 6B). Co‐IP assays showed that c‐Cbl primarily interacts with the EGFR's C‐terminal (CT) domain of EGFR (Figure S7D). Previous reports indicate that c‐Cbl binds EGFR at tyrosine 1045 (Tyr1045) to mediate ubiquitination and degradation [26, 40, 41], and indeed, we found that a Tyr1045 mutant significantly reduced c‐Cbl's interaction with EGFR (Figure S7E) and its ability to mediate EGFR ubiquitination (Figure 6C).

**FIGURE 6:**
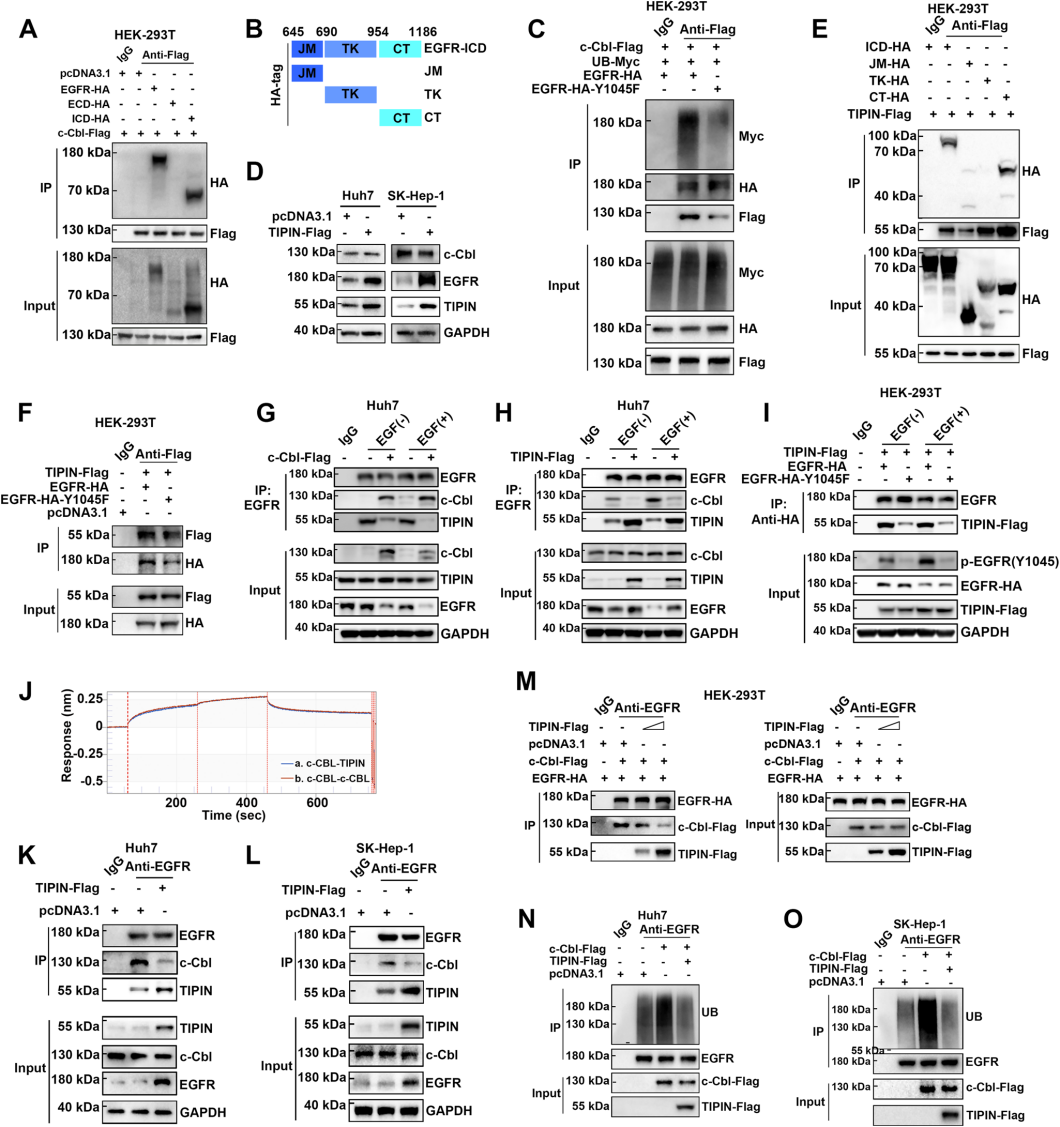
TIPIN binds to EGFR and suppresses c‐Cbl‐dependent EGFR ubiquitination and degradation. (A) IP assays evaluating interactions between distinct EGFR truncations and c‐Cbl. (B) Schematic of EGFR ICD truncations generated based on UniProt domain annotations. (C) Ubiquitination assays revealed that the Tyr1045 mutation in the ICD impairs c‐Cbl‐dependent EGFR ubiquitination. (D) Protein expression levels of c‐Cbl and EGFR in liver cancer cells with forced TIPIN overexpression were examined via immunoblot assays. (E) IP assays assessing interactions between TIPIN and EGFR ICD truncations. (F) IP assays showing TIPIN binding to EGFR Tyr1045. (G‐I) Co‐immunoprecipitation assays evaluating the interactions among EGFR, c‐Cbl, and TIPIN under EGF‐stimulated or unstimulated conditions. (G, H) Co‐IP analysis in Huh7 cells transfected with indicated constructs, with or without EGF treatment. (I) Co‐IP comparing the binding affinity of TIPIN to wild‐type EGFR and EGFR‐Y1045F mutant in HEK‐293T cells. (J) Protein‐protein affinity assay demonstrating competitive binding of TIPIN and c‐Cbl to EGFR. (K–M) TIPIN overexpression modulates both endogenous (K, L) and exogenous (M) c‐Cbl‐EGFR interactions. (N, O) Ubiquitination assays demonstrated that TIPIN overexpression inhibited c‐Cbl‐dependent ubiquitination of EGFR in liver cancer cells.

We evaluated whether TIPIN interferes with c‐Cbl‐dependent EGFR ubiquitination using an overexpression setup. Increased TIPIN raised EGFR protein amounts, whereas c‐Cbl expression remained unaltered (Figure 6D). Co‐IP assays showed that TIPIN interacts with the EGFR's CT domain (Figure 6E). Furthermore, the Y1045F mutation attenuated TIPIN binding to EGFR (Figure 6F). We next sought to distinguish whether TIPIN‐EGFR association relies on ligand‐triggered phosphorylation at Tyr1045, rather than just the presence of this amino acid residue. Co‐IP assays with or without EGF stimulation revealed that EGF treatment robustly strengthened the binding of c‐Cbl to EGFR, whereas the TIPIN‐EGFR interaction was not markedly enhanced upon EGF stimulation (Figure 6G,H). Nevertheless, the Y1045F mutation partially attenuated TIPIN association with EGFR (Figure 6I). These data suggest TIPIN can form complexes with EGFR without ligand‐triggered EGFR activation, while Tyr1045 strengthens this physical contact and acts as a key interaction interface.

BLI assays demonstrated that TIPIN competes with c‐Cbl for binding to EGFR (Figure 6J). Co‐IP experiments further showed that TIPIN overexpression markedly reduced the association between c‐Cbl and EGFR (Figure 6K,L) and progressively decreased c‐Cbl's binding affinity for EGFR (Figure 6M). Ectopic c‐Cbl expression restored EGFR ubiquitination in TIPIN‐overexpressing Huh7 cells (Figure S7F), indicating that elevated c‐Cbl could counteract the inhibitory effect of TIPIN. Ubiquitination assays confirmed that TIPIN overexpression significantly suppressed c‐Cbl‐mediated EGFR ubiquitination (Figure 6N,O).

Our results support this refined competitive mechanism: TIPIN binds unphosphorylated EGFR, whereas c‐Cbl recruitment depends heavily on phosphorylated Tyr1045. Following EGF treatment, elevated p‐Tyr1045 creates a preferred binding site for c‐Cbl. TIPIN competes with c‐Cbl at this phosphorylated residue, blocking c‐Cbl‐dependent ubiquitination and the subsequent lysosomal breakdown of EGFR. Such competition preserves EGFR protein abundance and maintains its downstream oncogenic signaling in liver cancer cells.

### 3.7 Suppression of TIPIN Expression Inhibits Liver Cancer ECM Remodeling and Angiogenesis

GO enrichment analysis indicated that TIPIN was primarily associated with the extracellular region (Figure 7A). Further gene set enrichment analysis (GSEA) demonstrated that loss of TIPIN altered signaling related to cell‐matrix adhesion and angiogenesis (Figure 7B,C). ECM remodeling driven by CAFs enhances tumor cell motility and invasion [42]. To dissect the crosstalk connecting TIPIN, CAFs, and ECM, we established a co‐culture model of Hepa 1–6/CAF cells. The identity of CAFs used in co‐culture assays was validated by flow cytometry (Figure S8A–H). TIPIN knockdown in this system notably dampened the motility and invasiveness of CAFs (Figure 7D). Angiogenesis assays using co‐cultured liver cancer cells and HUVECs revealed that depletion of TIPIN impaired angiogenic processes (Figure 7E). Masson's trichrome staining showed reduced collagen in TIPIN‐knockdown subcutaneous tumors (Figure 7F). IHC analysis further revealed decreased levels of α‐smooth muscle actin (α‐SMA, a CAF marker) and CD31 (an angiogenesis marker) in these tumors (Figure 7F). Reciprocal rescue assays confirmed that EGFR silencing abrogated the promotive effects of TIPIN overexpression on CAFs motility and HUVECs tube formation (Figure 7G,H), verifying that TIPIN drives stromal remodeling mainly via the TIPIN‐EGFR axis.

**FIGURE 7:**
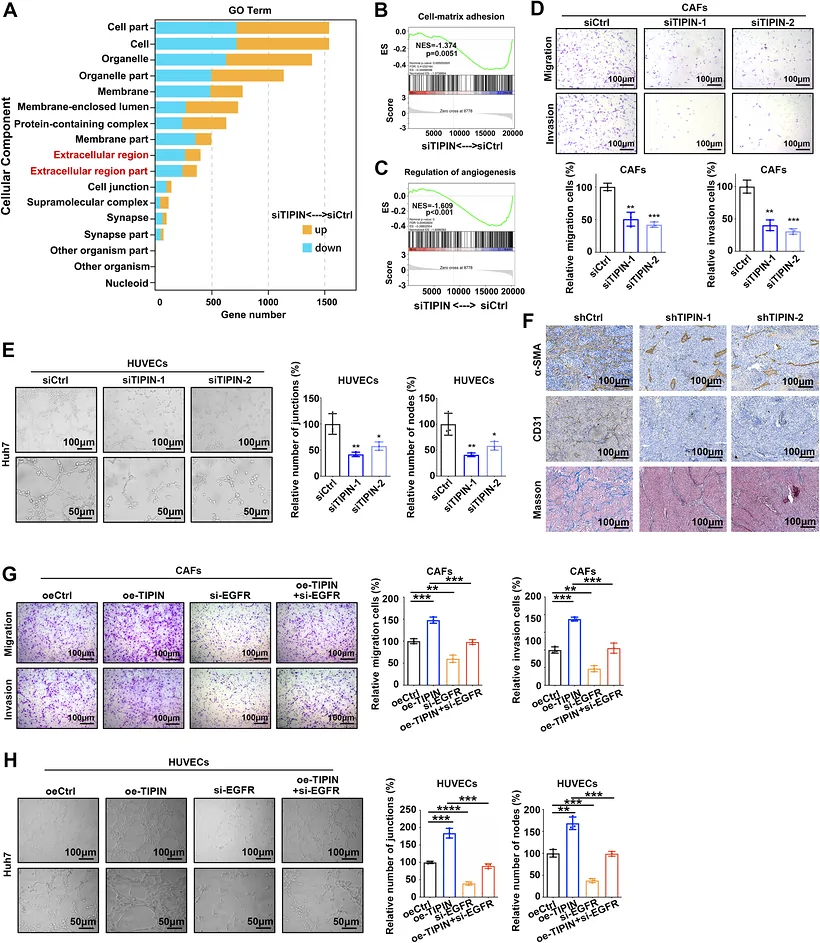
TIPIN knockdown suppresses angiogenesis and ECM remodeling. (A) GO enrichment analysis indicates a strong association of TIPIN with the extracellular region. All differential expression analyses were performed as siTIPIN vs. siCtrl. (B, C) GSEA results of “cell‐matrix interaction” and “regulation of angiogenesis” pathways. (D) Cell co‐culture system used to explore TIPIN's effect on CAF activity. Transwell assays confirmed that CAF activity was inhibited after TIPIN knockdown (mean ± SD, unpaired Student's t‐test). (E) A HUVEC tube formation assay was applied to validate the inhibitory effect of TIPIN silencing on angiogenesis. Histograms represent relative numbers of junctions and nodes (mean ± SD, unpaired Student's t‐test). (F) Xenograft tumor specimens from TIPIN‐knockdown and control groups were processed for Masson's trichrome staining and IHC detection of α‐SMA and CD31. (G) Transwell migration and invasion assays of CAFs treated with conditioned medium from Huh7 cells with indicated treatments (oeCtrl, oe‐TIPIN, si‐EGFR, oe‐TIPIN + si‐EGFR). Scale bar, 100 µm. (H) HUVEC tube formation assays incubated with conditioned medium from Huh7 cells under the indicated genetic manipulations. Scale bar, 100 µm. Quantitative statistics are shown on the right. Data are presented as mean ± SD. ^**^ *p* < 0.01, ^***^ *p* < 0.001, ^****^ *p* < 0.0001.

### 3.8 TIPIN Depletion Sensitizes Liver Cancer to Lenvatinib‐Based Therapy

For patients with advanced liver cancer, lenvatinib is a standard first‐line agent, and prior evidence indicates that EGFR‐directed therapy can boost its anti‐tumor activity [13, 31, 43]. Activation of the MEK/ERK pathway—downstream of EGFR—reduces lenvatinib sensitivity [44]. Short‐term Lenvatinib incubation significantly elevated TIPIN and EGFR protein abundance in a dose‐ and time‐dependent fashion, indicating that lenvatinib stimulation directly promotes TIPIN expression as an adaptive cellular response (Figure S9A,B). We hypothesized that TIPIN knockdown‐induced EGFR downregulation may sensitize liver cancer cells to lenvatinib. First, TIPIN knockdown consistently lowered the half‐maximal inhibitory concentration (IC_50_) of lenvatinib (Figure 8A). Additional functional assays, including colony formation, cell adhesion, apoptosis, tumorsphere formation, and transwell assays, demonstrated that combining TIPIN knockdown with lenvatinib produced significantly greater antitumor effects than lenvatinib alone (Figure 8B–E; Figure S9C–F). Tumors in the TIPIN‐knockdown plus lenvatinib group displayed markedly reduced volumes and weights compared with other groups (Figure 8F–I). Lenvatinib treatment reduced the phosphorylation of STAT3 and ERK1/2, and simultaneously induced compensatory accumulation of EGFR and p‐EGFR. TIPIN knockdown eliminated the EGFR upregulation triggered by lenvatinib. The combined treatment of TIPIN silencing and lenvatinib had a stronger inhibitory effect on STAT3 and ERK1/2 signaling, thereby stimulating synergistic anti‐tumor activity (Figure 8J; Figure S9G).

**FIGURE 8:**
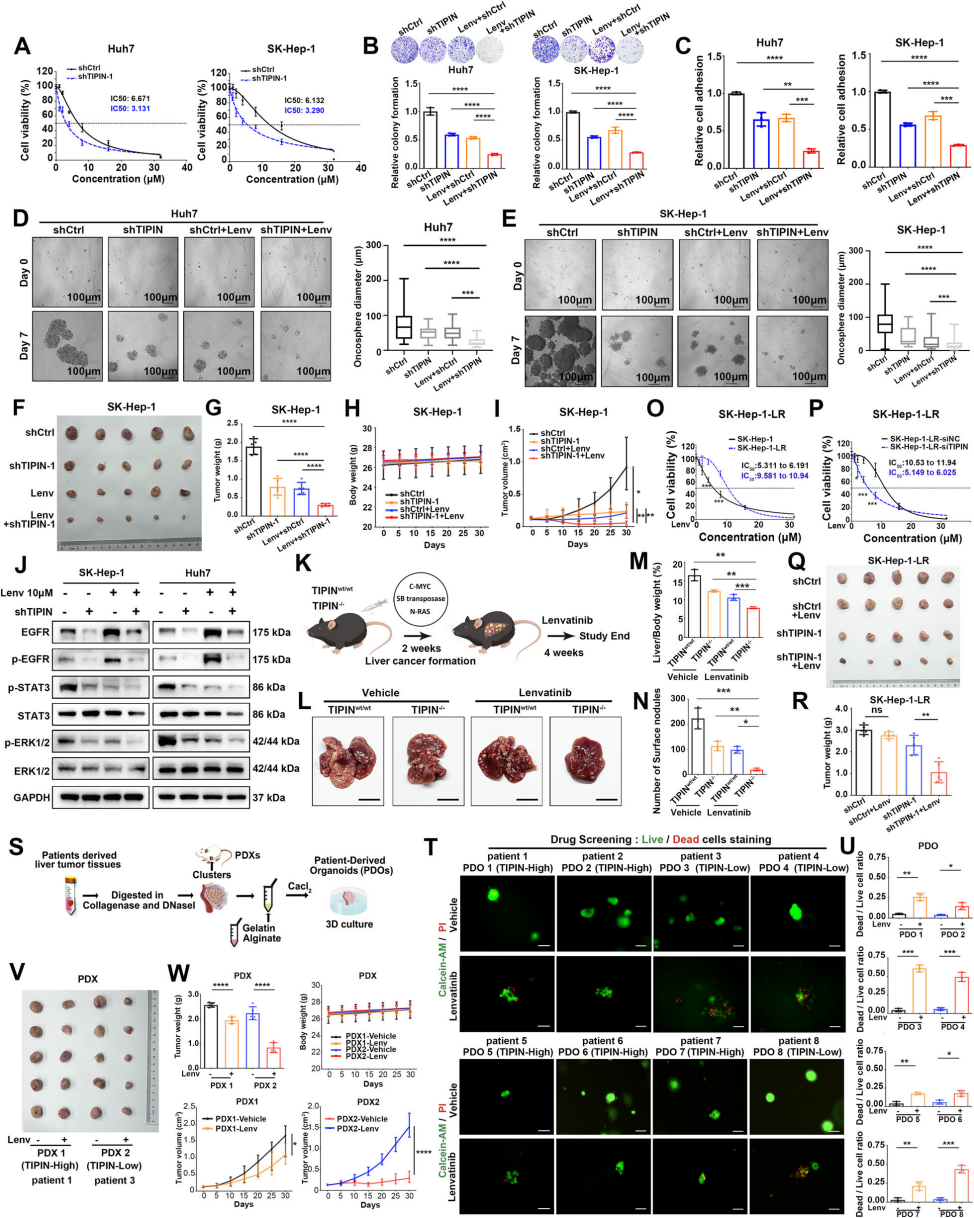
TIPIN knockdown sensitizes liver cancer cells to lenvatinib. (A) Dose‐response curve of the indicated cell lines treated with lenvatinib from a 2‐day CCK‐8 assay. (B) Colony formation assays confirmed that TIPIN silencing markedly strengthens the suppressive effect of lenvatinib on tumor cell proliferation (*n* = 3 per group; mean ± SD, unpaired Student's t‐test). (C) Adhesion functional assays demonstrated that TIPIN knockdown and lenvatinib synergistically inhibit liver cancer cell adhesion (mean ± SD, unpaired Student's t‐test). (D, E) Oncosphere formation assays of liver cancer cells under four different conditions: TIPIN‐knockdown, lenvatinib, TIPIN‐knockdown + lenvatinib, and control groups (scale bar, 2 µm). (F–I) The photograph of subcutaneous tumors in the indicated groups. Statistical analysis of tumor weight (g), tumor volume (mm^3^), and mouse body weight in SK‐Hep‐1 xenograft models (*n* = 5 per group, mean ± SD, unpaired Student's t‐test). (J) Immunoblot analysis illustrating protein expression profiles in cells under the specified treatment conditions. (K) Flowchart of the HDI models of TIPIN knockout mice after lenvatinib treatment. (L) Representative images of tumor lesions in the HDI models. (M, N) Liver weight/body weight ratio and number of surface nodules in HDI models with the indicated treatments (mean ± SD, unpaired Student's t‐test). (O) Cell proliferation assays were conducted to determine the lenvatinib IC_50_ values in SK‐Hep‐1 and lenvatinib‐resistant SK‐Hep‐1‐LR cells (*n* = 6 each group). (P) Cell proliferation assay showing that knockdown of TIPIN in SK‐Hep‐LR cells could reverse lenvatinib resistance. (Q, R) In vivo xenograft assays using SK‑Hep‑1‑LR lenvatinib‑resistant cells. (Q) Photographs of subcutaneous tumors in the indicated groups. (R) Tumor weight (g) of SK‐Hep‐1‑LR xenograft models in different treatment groups (*n* = 5 per group, mean ± SD, unpaired Student's t‐test). (S) Schematic illustrating the application of PDO and PDX models to verify drug responsiveness in patient samples with distinct TIPIN expression levels. (T, U) Live/dead cell staining assay in PDO models upon lenvatinib treatment. (T) Representative images of PI (red) and Calcein‐AM (green) staining in eight independent PDO samples under vehicle or lenvatinib exposure. Scale bars, 100 µm. (U) Quantification of dead/live cell ratio for each PDO sample after indicated treatments (mean ± SD, unpaired Student's t‐test). (V, W) PDX models demonstrate that tumors with low TIPIN expression exhibit superior lenvatinib therapeutic response. (V) Representative photographs of harvested PDX tumors from TIPIN‐high (PDX1, patient 1) and TIPIN‐low (PDX2, patient 3) groups under vehicle or lenvatinib treatment. (W) Quantification of endpoint tumor weight, mouse body‐weight monitoring, and tumor‐volume growth curves for PDX1 and PDX2 (mean ± SD; *n* = 5 per group).

To further assess the role of TIPIN in lenvatinib responsiveness, we established HDI‐induced liver cancer models in *TIPIN* ^−^ *^/^* ^−^ and *TIPIN^wt/wt^* mice prior to lenvatinib administration (Figure 8K). The combination of TIPIN knockout and lenvatinib has the strongest inhibitory effect on tumor progression, indicating that TIPIN is an important factor in sensitivity to lenvatinib (Figure 8L–N).

We next generated lenvatinib‐resistant liver cancer cell lines to explore TIPIN's role in lenvatinib resistance (Figure 8O and Figure S10A). These resistant cells showed increased expression of TIPIN, EGFR, and ERK pathway components (Figure S10B,C). TIPIN knockdown reversed these molecular changes and restored sensitivity to lenvatinib, as demonstrated by proliferation and colony formation assays (Figure 8P; Figure S10D–F). To further validate these findings in vivo, we established subcutaneous xenografts using lenvatinib‐resistant SK‐Hep‐1‐LR cells. TIPIN knockdown suppressed tumor growth and restored sensitivity to lenvatinib (Figure 8Q,R; Figure S10G–I).

We next examined whether intrinsic TIPIN levels could serve as predictive biomarkers for lenvatinib efficacy using PDOs [45, 46] and PDX models [45, 47] previously established in our laboratory (Figure 8S). PDOs from low‐TIPIN tumors were markedly more sensitive to lenvatinib than those from high‐TIPIN tumors (Figure S10J,K and Figure 8T,U). Similarly, PDX models derived from low‐TIPIN patients responded robustly to lenvatinib, whereas those derived from high‐TIPIN patients exhibited limited responsiveness (Figure 8V,W). In conclusion, these findings suggest a correlation between high endogenous TIPIN expression and poor lenvatinib response. TIPIN may represent a promising biomarker and therapeutic target for optimizing treatment responses in liver cancer.

## 4 Discussion

In the present study, we systematically and comprehensively investigated the role of TIPIN in liver cancer proliferation and metastasis. Our experimental evidence identifies TIPIN as a novel oncogenic driver in liver cancer, with its aberrant expression tightly controlled by DNA epigenetic modification. Mechanistically, TIPIN exerts pro‐tumor effects by competing with c‐Cbl: this competition interferes with EGFR ubiquitination, thereby preserving EGFR protein stability and ultimately driving liver cancer cell proliferation and metastasis. Accordingly, targeting the TIPIN‐EGFR signaling axis may provide a promising strategy to enhance lenvatinib sensitivity and restrain tumor progression in clinical liver cancer treatment.

Bioinformatic analysis of TCGA and GEO datasets revealed significant correlations between TIPIN expression and DNA methylation status. As a major epigenetic regulatory mechanism, DNA methylation alters gene transcription without changing genomic DNA sequences [48]. Aberrant methylation is prevalent in various human malignancies, including liver cancer, and preferentially occurs at CpG island loci [49]. Our study identified the UR_CpG island located in the TIPIN promoter region, which exhibits significant hypomethylation in liver cancer tissues—consistent with TCGA data. Methyl‐target sequencing results confirmed that DNA methylation governs endogenous TIPIN expression, with DNMT1 acting as the primary methyltransferase responsible for this epigenetic regulation. Nevertheless, the function of TIPIN in liver cancer proliferation and metastasis remained poorly understood. Our data indicate that TIPIN levels are markedly increased in liver tumor tissues relative to non‐tumor samples, and this upregulation correlates with poorer patient outcomes. Moreover, functional assays confirmed that TIPIN markedly promotes liver cancer proliferation and metastasis. Classically, TIPIN forms a complex with TIMELESS to safeguard replication forks, and its upregulation promotes tumor proliferation, survival, and genomic instability in diverse cancers [32, 33, 34, 35]. Our IP‐MS also successfully captured well‐documented TIPIN interactors, including MCM2, MCM3, and MCM4 [34], confirming the reliability and specificity of our experimental system. Importantly, EGFR was identified as a previously unrecognized TIPIN‐binding protein in our mass spectrometry results. We further validated this physical interaction via sequential co‐IP assays, identifying a novel TIPIN–EGFR regulatory axis that expands the known functional repertoire of TIPIN beyond DNA replication control.

EGFR is well‐established as an oncogenic driver in breast cancer, non‐small cell lung cancer, and other tumor types, and approximately 66% of liver cancer tissues exhibit moderate or abundant EGFR protein expression [50]. EGFR‐driven signaling activity is tightly associated with the progression of liver cancer [51]. Rescue experiments verified that TIPIN promotes liver cancer proliferation and metastasis by upregulating EGFR signaling. IP analyses revealed that TIPIN physically interacts with the EGFR's ICD. Ubiquitination is a critical post‐translational modification that can regulate the expression and activity of key biological proteins [52]; its dysregulation contributes to liver cancer development and progression [44, 53]. Ubiquitination experiments revealed that TIPIN reduces EGFR ubiquitination, keeping EGFR protein levels stable.

C‐Cbl, a RING‐type E3 ubiquitin ligase widely expressed in cells, takes part in intracellular signal transmission [54]. As a well‐characterized E3 ligase specific for EGFR, c‐Cbl triggers EGFR internalization and protein breakdown [55, 56]. Published work has demonstrated that c‐Cbl binds the Tyr1045 residue on the intracellular domain of EGFR to drive its ubiquitination [26]. Co‐IP data further revealed that mutation of Tyr1045 weakens c‐Cbl‐EGFR binding, which attenuates c‐Cbl‐dependent ubiquitination and degradation of EGFR. No previous reports have addressed the functional link between TIPIN and c‐Cbl. Notably, our IP results showed that TIPIN—like c‐Cbl—binds to ICD Tyr1045. These findings indicate that TIPIN binds EGFR and weakens c‐Cbl‐triggered EGFR ubiquitination and degradation, thereby sustaining EGFR signaling activation. Mechanistically, c‐Cbl predominantly catalyzes K63‐linked ubiquitination of activated EGFR to trigger receptor endocytosis and lysosomal turnover, rather than K48‐linked ubiquitination and proteasomal degradation [57, 58]. Competitive binding of TIPIN impedes this K63 ubiquitination cascade and blocks downstream EGFR clearance.

As an orally available multi‐kinase inhibitor against VEGFR, FGFR, and PDGFR, lenvatinib blocks tumor angiogenesis and cell proliferation [59] and is commonly applied for liver cancer therapy [60]. However, acquired drug resistance frequently curtails its clinical efficacy [59, 61]. Earlier work has shown that EGFR levels rise markedly in liver cancer cells after lenvatinib exposure [13, 31, 62]. In this context, our epigenetic results raise an intriguing translational hypothesis: TIPIN promoter methylation level may represent a candidate predictive biomarker for lenvatinib response in liver cancer. Tumors featuring TIPIN promoter hypermethylation display suppressed TIPIN expression, facilitating c‐Cbl‐driven EGFR ubiquitination and degradation. As persistent EGFR signaling promotes lenvatinib resistance, liver cancer with high TIPIN promoter methylation and low TIPIN abundance may achieve superior therapeutic outcomes. Formal verification of this correlation requires matched clinical samples with TIPIN methylation detection and longitudinal lenvatinib follow‐up data, which we plan to explore in follow‐up research. We therefore investigated whether TIPIN knockdown‐induced EGFR downregulation plays a role in enhancing lenvatinib sensitivity. These results strongly support our hypothesis that TIPIN knockdown sensitizes liver cancer cells to lenvatinib via suppression of EGFR‐dependent signaling. Given that lenvatinib exerts anti‐tumor effects by targeting multiple RTKs including VEGFR, FGFR and PDGFR, we experimentally tested whether TIPIN could regulate these additional lenvatinib targets. As shown in Figure S11, endogenous co‐IP assays failed to detect physical interaction between TIPIN and MET, FGFR2, VEGFR2, or PDGFRβ. Consistently, siRNA‐mediated TIPIN knockdown did not alter the steady‐state protein abundance of these RTKs in hepatocellular carcinoma cells. These data support that TIPIN‐dependent receptor stabilization is not a broad, general effect for all RTKs, but exhibits specificity toward EGFR in liver cancer cells.

Nevertheless, the present study has several limitations that deserve consideration. First, we found that TIPIN expression is upregulated following lenvatinib treatment; further investigation into the underlying mechanism in subsequent studies may deepen our understanding of TIPIN's role in lenvatinib resistance. Second, the majority of experiments in the present work were performed in parallel across two cell models. Huh7, a well‐validated hepatocellular carcinoma (HCC) cell line, served as the primary model to underpin our core mechanistic and functional conclusions, while SK‐Hep‐1 was included as a supplementary liver cancer‐related cell model to cross‐validate the observations. As documented in published literature, SK‐Hep‐1 originates from hepatic sinusoidal endothelial cells rather than hepatocytes. Accordingly, findings from SK‐Hep‐1 may reflect broader biological features of liver cancer rather than canonical hepatocellular carcinoma phenotypes, and we have exercised appropriate caution in interpreting and describing these results throughout the manuscript. Follow‐up validation using additional well‐characterized HCC cell lines will be conducted to further strengthen our conclusions. Third, genetic manipulation of PDOs is technically challenging, so we did not perform TIPIN knockdown in individual PDO lines. Our PDO/PDX data only demonstrate a correlative association between endogenous TIPIN expression and lenvatinib sensitivity, not formal causal evidence. Future genetic modification of PDOs will help validate TIPIN's causal function in patient‐derived tumor models. Fourth, the tumor immune microenvironment has emerged as a key focus in research on tumor progression and drug resistance in recent years, yet its interplay with TIPIN in regulating liver cancer proliferation, metastasis, and therapeutic response remains unaddressed in the present study—representing a promising direction for future exploration. Fifth, we currently lack clinical or preclinical agents that specifically target TIPIN or its downstream signaling pathways. Notably, therapeutic strategies using nanocarrier‐encapsulated siRNA for targeted tumor therapy have been developed in recent years [63, 64], and this approach merits further exploration for TIPIN‐targeted liver cancer therapy.

## Author Contributions

X.J., S.Z., Y.T., and S.C. contributed equally to this work. X.J., S.Z., Y.T., S.C., Q.L., and P.S. designed and supervised the study. X.J., S.Z., Y.T., S.C., X.Z., J.C., S.B., L.Z., C.Z., and H.M. performed the experiment. X.J., S.C., W.S., C.J., and M.Z. performed the acquisition of the data and statistical analysis. All the authors drafted the manuscript. H.X., Q.L., and P.S. revised the final manuscript. All the authors have approved the final version of the manuscript.

## Conflicts of Interest

The authors declare no conflicts of interest.

## Supporting information




**Supporting File 1**: advs78638‐sup‐0001‐SuppMat.docx.

**Supporting File 2**: advs78638‐sup‐0002‐Tables S1‐S11.xlsx.

## Data Availability

The data that support the findings of this study are available from the corresponding author upon reasonable request.
