# Supplementary material for: TIPIN Couples Epigenetic Rewiring to EGFR Stabilization and Lenvatinib Resistance in Liver Cancer
Source: Adv Sci (Weinh). 2026 Nov 9:e78638. Online ahead of print. doi: 10.1002/advs.78638 (PMC13749002; doi:10.1002/advs.78638)
Supplement: Supplementary file 1 [file ADVS-9999-e78638-s002.docx]

**Supplementary Materials**

**Title:** TIPIN couples epigenetic rewiring to EGFR stabilization and lenvatinib resistance in liver cancer

**Author**

**Xing Jia**^1,2,5,6,7#^, **Shiyu Zhang**^3,5,6,7#^, **Yangbo Tang**^1,5,6,7#^, **Shinuo Chen**^1,5,6,7#^, Xingxin Zhu^4,5,6,7^, Junran Chen^1,5,6,7^, Suchen Bian^1,5,6,7^, Lei Zheng^1,5,6,7^, Chi Zhang^1,5,6,7^, Haojing Ma^1,5,6,7^, Cheng Jin^1,5,6,7^, Mengqiao Zhou^1,5,6,7^, Haiyang Xie^1,5,6,7^, **Qinglin Li^8*^**, and **Penghong Song^1,5,6,7,9*^**

**Affiliations**

1. Division of Hepatobiliary and Pancreatic Surgery, Department of Surgery, The First Affiliated Hospital, Zhejiang University School of Medicine, Hangzhou 310003, China

2. Thyroid Surgery Department, Zhejiang Cancer Hospital, Hangzhou 310022, Zhejiang, China.

3. Department of Thoracic Surgery, Second Affiliated Hospital, Zhejiang University School of Medicine, 88 Jiefang Road, Shangcheng District, Hangzhou, China.

4. Department of Thoracic Surgery, The First Affiliated Hospital of Zhejiang University School of Medicine, Hangzhou, China

5. NHC Key Laboratory of Combined Multi-organ Transplantation, Hangzhou 310003, China

6. Key Laboratory of the Diagnosis and Treatment of Organ Transplantation, Research Unit of Collaborative Diagnosis and Treatment for Hepatobiliary and Pancreatic Cancer, Chinese Academy of Medical Sciences (2019RU019), Hangzhou 310003, China

7. Key Laboratory of Organ Transplantation, Research Center for Diagnosis and Treatment of Hepatobiliary Diseases, Zhejiang Province, Hangzhou 310003, China

8. Tongde Hospital of Zhejiang Province Affiliated to Zhejiang Chinese Medical University, Zhejiang Province, Hangzhou 310003, China

9. State Key Laboratory for Diagnosis and Treatment of Infectious Diseases, First Affiliated Hospital, Zhejiang University School of Medicine, Hangzhou 310003, China

**#These authors contributed equally to this work.**

***Corresponding author:**

Penghong Song, The First Affiliated Hospital Zhejiang University, 79 Qingchun Road, Hangzhou 310003, China, Email: [songpenghong@zju.edu.cn](mailto:songpenghong@zju.edu.cn); and Qinglin Li, Tongde Hospital of Zhejiang Province Affiliated to Zhejiang Chinese Medical University, Zhejiang Province, Hangzhou 310003, China, E-mail: qinglin200886@126.com.

**Supplementary Figure S1. Baseline liver phenotypes of TIPIN^-/-^ mice and TCGA TIPIN methylation analysis in LIHC**
(A) Representative macroscopic liver photographs and HE staining of liver sections from adult TIPIN^wt/wt^ and TIPIN^-/-^ mice. No obvious histological abnormalities are observed in TIPIN^-/-^ livers (scale bar, 100 μm).

(B) Quantification of serum ALT and AST levels in naive TIPIN^wt/wt^ and TIPIN^-/-^ mice. No significant differences were detected between the two genotypes (ns, not significant; unpaired Student’s t-test).

(C) TIPIN is hypomethylated in liver cancer (LIHC; TCGA database).

(D) Relative methylation level of TIPIN probes (cg04792910, cg18310110, cg24360197, cg11797222, cg02298012 and cg22492020) in liver cancer patients in TCGA databases (mean ± SD, unpaired Student’s t-test).

(E) Pearson correlation between methylation level and TIPIN mRNA level (TCGA database).

(F) The probe cg1164506 is hypomethylated in liver cancer (LIHC; TCGA database).

**Supplementary Figure S2. Analysis of TIPIN promoter CpG methylation and endogenous protein expression in liver cancer.**

(A) Prediction of unreported CpG islands in the TIPIN promoter region using the EMBOSS website.

(B) DNA methylation sequencing of 10 pairs of tumor and adjacent tissues (Cohort 5) at the 11 designed probe sites of the TIPIN UR_CpG island (mean ± SD, paired Student’s t-test).
(C, D) Relative quantification results of immunoblotting in Figure 2I and Figure 2L (means ± SD, unpaired Student’s t-test).

(E) Immunoblot analysis showing endogenous TIPIN protein abundance in a panel of human liver cancer cell lines.

**Supplementary Figure S3. Downregulation of TIPIN significantly curtailed the expansion, dispersal, and invasive behavior of live cancer cells.**

(A) Oncosphere formation assays of SK-Hep-1 cells in the shTIPIN and shCtrl groups (mean ± SD, unpaired Student’s t-test).

(B) Transwell assay showing the migration and invasion capacity of SK-Hep-1 cells infected with TIPIN siRNA or siCtrl (n = 3 each group). Representative images (Left) and relative quantification (Right) are displayed. Scale bars, 100 μm.

(C) Wound healing assays were conducted to assess the motility of SK-Hep-1 cells in the indicated treatment groups at 0 and 24 h. Representative images (Left) and relative quantification (Right) are shown (n = 3 each group, mean ± SD, unpaired Student’s t-test).

(D-I) Cell Adhesion Kit and trypsin digestion assays confirming TIPIN knockdown inhibits cell adhesion, while TIPIN overexpression increases cell adhesion (mean ± SD, unpaired Student’s t-test).

(J) Flowchart of RNA sequencing of TIPIN-knockdown Huh7 cells and control group.

(K) Principal component analysis (PCA) of Huh7 cells in each group (orange = siCtrl; blue = siTIPIN).

(L, M) RNA sequencing showing changes in transcription levels of TIPIN-knockdown and control SK-Hep-1 cells. A total of 626 differentially expressed genes were screened (DESeq2; FDR < 0.05, |Log₂FC| > 1.2).

(N, O) Gene set enrichment analysis (GSEA) results of the “Focal adhesion” and “Regulation of actin filament-based movement” pathway in TIPIN-knockdown and control liver cancer cells.

(P) Relative quantification results of immunoblotting in Figure 3O (means ± SD, unpaired Student’s t-test).

**Supplementary Figure S4. EGFR expression correlates with TIPIN levels and TIPIN facilitates tumor progression via EGFR regulation.**

(A) Relative quantification results of immunoblotting in Figure 5D (means ± SD, unpaired Student’s t-test).

(B) Immunoblot analysis of EGFR and its downstream signaling proteins in liver cancer cells with TIPIN overexpression. Bar graphs show relative protein quantification normalized to GAPDH (mean ± SD; unpaired Student’s t‑test).

(C) Ectopic EGFR expression rescues the inhibitory effects of TIPIN knockdown on cell proliferation (mean ± SD, unpaired Student’s t-test).

(D-F) Ectopic EGFR expression rescues the inhibitory effects of TIPIN knockdown on oncosphere formation (D), adhesion (E), and metastasis (F) in liver cancer cells (mean ± SD, unpaired Student’s t-test).

(G) Schematic illustration of full-length TIPIN (WT) and two truncation mutants. Immunoblot analysis of EGFR, p-ERK1/2 and total ERK1/2 expression in shTIPIN SK-Hep-1 cells reconstituted with empty vector, Flag-TIPIN-WT, Flag-TIPIN-Δ1 or Flag-TIPIN-Δ2.

(H) Representative images and quantitative statistics of colony formation assays in the indicated cell groups (mean ± SD, unpaired Student’s t-test).

(I) Quantification of cell adhesion capacity upon TIPIN knockdown and rescue with different TIPIN constructs (mean ± SD, unpaired Student’s t-test).

**Supplementary Figure S5. TIPIN regulated surface EGFR expression levels in liver cancer cells.**

(A) Flow cytometric and quantification of residual surface EGFR after EGF stimulation. Surface EGFR geometric mean fluorescence intensity (Geo-MFI) at 15 min post-EGF was normalized to t = 0 baseline within each group (mean ± SD, unpaired Student’s t-test).

(B) Immunoblot analysis of p‑EGFR, total EGFR and TIPIN in TIPIN-knockdown Huh7 and SK-Hep-1 liver cancer cells following EGF stimulation for the indicated time points. Quantifications show relative p-EGFR normalized to GAPDH, relative total EGFR normalized to GAPDH, and receptor-normalized p-EGFR (p-EGFR/total-EGFR). Data represent mean ± SD from three independent biological replicates.

**Supplementary Figure S6. TIPIN maintained EGFR stability by inhibiting lysosomes.**

(A) TIPIN knockdown shortens EGFR half-life. Huh7 cells were transfected with lentiviral vectors and treated with cycloheximide (CHX, 10 μg/mL) for the indicated durations. Quantification of EGFR protein stability is shown (B).

(B) Relative quantification results of immunoblotting in Figure 5L (means ± SD, unpaired Student’s t-test).

(C) Immunoblot analysis of EGFR expression in TIPIN-knockdown liver cancer cells with or without MG132 treatment. Quantification of relative TIPIN and EGFR protein levels normalized to GAPDH are shown below (means ± SD, unpaired Student’s t-test).

(D-E) Ubiquitination analysis of EGFR in liver cancer cells with TIPIN knockdown (D) or overexpression (E).

**Supplementary Figure S7. TIPIN inhibits c-Cbl-mediated ubiquitination of EGFR.**

(A) Upstream E3 ubiquitin ligases of EGFR were analyzed using the UbiBrowser database.

(B) STRING database analysis revealing the top 10 candidate interacting proteins of EGFR.

(C) Ubiquitination assays for c-Cbl-mediated EGFR ubiquitination of Huh7 and SK-Hep-1 cells.

(D) IP assays evaluating interactions between distinct EGFR-ICD truncations and c-Cbl in HEK-293T cells.

(E) IP assays showing c-Cbl binding to EGFR Tyr1045.

(F) Co-IP assay detecting EGFR ubiquitination upon c-Cbl overexpression in Huh7 cells stably overexpressing TIPIN.

**Figure S8. Flow cytometric characterization of immortalized mouse liver CAFs.**

(A) FSC-A/SSC-A plot showing gating of the main intact cell population.

(B) FSC-A/FSC-H plot to identify single cells and remove cell doublets.

(C) Live cells were selected using live/dead (LD) staining.

(D) CD45-positive immune cells were excluded from the live single-cell population.

(E) CD31-positive endothelial cells and CD326 (EpCAM)-positive epithelial cells were further excluded.

(F–H) Representative histograms/dot plots showing expression of CAF markers α-SMA (F), FAP (G), and PDGFRα (H) in the CD45⁻CD31⁻CD326⁻ cell population. Percentages of marker-positive cells within each gate are displayed in each panel.

**Supplementary Figure S9. TIPIN knockdown sensitizes hepatocellular carcinoma cells to lenvatinib.**

(A) Immunoblot analysis of EGFR and TIPIN expression in SK-Hep-1 and Huh7 cells treated with increasing concentrations of lenvatinib (0, 5, 10 μM). Corresponding quantitative densitometry is shown on the right; GAPDH served as loading control (n = 3 biological replicates, mean ± SD).

(B) Time-course immunoblot analysis for EGFR and TIPIN in SK-Hep-1 and Huh7 cells after 5 μM lenvatinib treatment for 0, 12 and 24 h. Quantitative densitometry of protein levels is displayed (n = 3 biological replicates, mean ± SD).

(C, D) Flow-cytometric Annexin-V/PI apoptosis analysis in sh-Ctrl and sh-TIPIN Huh7 (C) and SK-Hep-1 (D) cells with or without 5 μM lenvatinib treatment. Quantification of apoptotic cell percentages is shown (n = 3 biological replicates per group, mean ± SD).

(E, F) Transwell migration (E) and invasion (F) assays for sh-Ctrl and sh-TIPIN HCC cells with or without lenvatinib exposure. Representative microscopic images and quantitative cell counts are presented. Scale bars, 100 μm (n = 3 biological replicates per group, mean ± SD).

(G) Relative quantification results of immunoblotting in Figure 8J (means ± SD, unpaired Student’s t-test).

**Supplementary Figure S10. TIPIN knockdown reversed lenvatinib resistance in lenvatinib resistant liver cancer cells.**

(A) Colony formation assay showing the effect of lenvatinib (5 μM) on SK-Hep-1 and SK-Hep-1-LR cells. Relative quantification is shown (n = 3 per group, mean ± SD, unpaired Student’s t-test).

(B, C) Immunoblot analysis (B) and corresponding quantitative densitometry (C) of TIPIN, EGFR, p‑ERK1/2, ERK1/2, p‑STAT3 and STAT3 in SK‑Hep‑1 and SK‑Hep‑1‑LR cells. GAPDH served as loading control.

(D, E) Immunoblot analysis (D) and quantitative densitometry (E) for the indicated proteins in SK‑Hep‑1‑LR cells after TIPIN siRNA‑mediated knockdown. GAPDH served as loading control (n = 3 biological replicates, mean ± SD).

(F) Colony‑formation assay showing that TIPIN depletion re‑sensitized SK‑Hep‑1‑LR cells to 5 μM lenvatinib. Relative colony‑formation quantification is presented (n = 3 biological replicates per group, mean ± SD, unpaired Student’s t‑test).

(G‑I) In‑vivo xenograft assays using SK‑Hep‑1‑LR lenvatinib‑resistant cells. (G) Immunoblot validation of TIPIN knockdown in SK‑Hep‑1‑LR stable cell lines. (H) Body‑weight monitoring of nude mice throughout the experiment. (I) Tumour‑volume growth curves of xenograft tumours (n = 5 mice per group, mean ± SD).

(J, K) Representative immunohistochemistry images (J) and immunoblot analysis (K) of TIPIN protein expression in human HCC clinical specimens. Scale bars, 100 μm. n = 8 independent patient samples.

**Supplementary Figure S11. TIPIN does not bind or modulate MET, FGFR2, VEGFR2 and PDGFRβ.**

(A) Endogenous co‑IP assays in Huh7 and SK‑Hep‑1 cells. Cell lysates were immunoprecipitated with anti‑TIPIN or control IgG, and immunoblotted for the indicated receptor tyrosine kinases and TIPIN.

(B) Immunoblot detection of MET, FGFR2, VEGFR2 and PDGFRβ after TIPIN knockdown by two distinct siRNAs in Huh7 and SK‑Hep‑1 cells. GAPDH was used as loading control. Quantification of relative protein levels normalized to GAPDH, mean ± SD, n=3 biological replicates. ****P < 0.0001; ns, no significance.
